# Is the middle Cambrian *Brooksella* a hexactinellid sponge, trace fossil or pseudofossil?

**DOI:** 10.7717/peerj.14796

**Published:** 2023-02-24

**Authors:** Morrison R. Nolan, Sally E. Walker, Tara Selly, James Schiffbauer

**Affiliations:** 1 Department of Geosciences, Virginia Polytechnic Institute and State University (Virginia Tech), Blacksburg, VA, United States of America; 2 Department of Geology, University of Georgia, Athens, GA, United States of America; 3 X-ray Microanalysis Core Facility, University of Missouri, Columbia, MO, United States of America; 4 Department of Geological Sciences, University of Missouri, Columbia, MO, United States of America

**Keywords:** Middle Cambrian, Hexactinellids, Sponges, Trace fossils, Pseudofossils, Concretions

## Abstract

First described as a medusoid jellyfish, the “star-shaped” *Brooksella* from the Conasauga shale Lagerstätten, Southeastern USA, was variously reconsidered as algae, feeding traces, gas bubbles, and most recently hexactinellid sponges. In this work, we present new morphological, chemical, and structural data to evaluate its hexactinellid affinities, as well as whether it could be a trace fossil or pseudofossil. External and cross-sectional surfaces, thin sections, X-ray computed tomography (CT) and micro-CT imaging, revealed no evidence that *Brooksella* is a hexactinellid sponge or a trace fossil. Although internally *Brooksella* contains abundant voids and variously orientated tubes consistent with multiple burrowing or bioeroding organisms, these structures have no relation to *Brooksella*’s external lobe-like morphology. Furthermore, *Brooksella* has no pattern of growth comparable to the linear growth of early Paleozoic hexactinellids; rather, its growth is similar to syndepositional concretions. Lastly, *Brooksella*, except for its lobes and occasional central depression, is no different in microstructure to the silica concretions of the Conasauga Formation, strongly indicating it is a morphologically unusual endmember of the silica concretions of the formation. These findings highlight the need for thorough and accurate descriptions in Cambrian paleontology; wherein care must be taken to examine the full range of biotic and abiotic hypotheses for these compelling and unique fossils.

## Introduction

Sponges that produce siliceous skeletons are the only benthic animals that secrete copious amounts of silica and are recently recognized as important sinks for biogenic silica and nutrient cycling in the oceans (Chu et al., 2011; Maldonado et al., 2011; Maldonado et al., 2021). The fossil record and molecular clock history of putative sponges extends back to the late Proterozoic, possibly to 890 million years, making them potentially among the first animals on Earth (*e.g.*, keratose demosponges; Turner, 2021), though many fossils of purported precambrian sponges are subject to significant controversy (Antcliffe, Callow & Brasier, 2014; Botting & Muir, 2018). The earliest fossils of partially biomineralized, siliceous spicules date to the early Cambrian or the latest Ediacaran, indicating either a later evolution of spicule biomineralization or a taphonomic bias against these structural components that are essential to sponge taxonomy prior to that time (Sperling et al., 2010; Chang et al., 2019; Tang et al., 2019).

Disarticulated and articulated sponge spicules are known from a variety of early-to-middle Cambrian Burgess Shale-type deposits (*e.g.*, Burgess Shale Lagerstätte of Canada and the earlier Series 2 Sirius Passet Lagerstätte of Greenland; Finks, 2003; Botting & Peel, 2016), though it was not until the middle Cambrian that the taxonomic affinities of these sponges become clearer. Most of these early and middle Cambrian sponges are preserved as compressions or impressions on shale with abundant spicules (Finks, 2003) except for an enigmatic star-shaped fossil interpreted as a hexactinellid sponge, *Brooksella alternata*, from the middle Cambrian Conasauga Lagerstätte of the southeastern US (northeastern Alabama, northwestern Georgia; Ciampaglio et al., 2006; Schwimmer & Montante, 2007). *Brooksella* is considered to have exceptional three-dimensional (3-D) preservation in chert concretions with radial morphology and numerous lobes (Ciampaglio et al., 2006). However, its identity has generated controversy since its discovery in the late 1800s by Charles Doolittle Walcott (Walcott, 1896; Walcott, 1898).

*Brooksella* was originally described by Walcott in 1896 as a jellyfish with tentacles, an umbrella (bell), and a gastric cavity. However, he also considered whether these medusoid forms were hexactinellid sponges despite finding no spicules or traces of spicules in his “large number” of thin sections of *Brooksella* (Walcott, 1898, his p. 21)—although he mentions finding a few hexactinellid-like spicule casts on the outer surface of non-medusoid concretions (Walcott, 1898, his p. 22). Since Walcott’s work, the taxonomic identity of *Brooksella* has been reevaluated many times (Table S1 and Fig. S1). The most recent reevaluation by Ciampaglio et al. (2006) suggests that *Brooksella* is a *Protospongia*-type reticulosan hexactinellid sponge (though later researchers have suggested that *Protospongia* specifically and reticulosans, and in general, are not hexactinellids, *e.g.*, Botting & Muir, 2018; Page, Butterfield & Harvey, 2009; and J Botting, pers. comm., 2022), which Walcott also suggested but later rejected, over a century earlier. As a consequence of this new taxonomic assignment, the Conasauga Formation is interpreted to be an exceptional fossil Lagerstätte with fossils preserved by extensive sponge-produced biogenic silica (Schwimmer & Montante, 2007).

**Figure 1:**
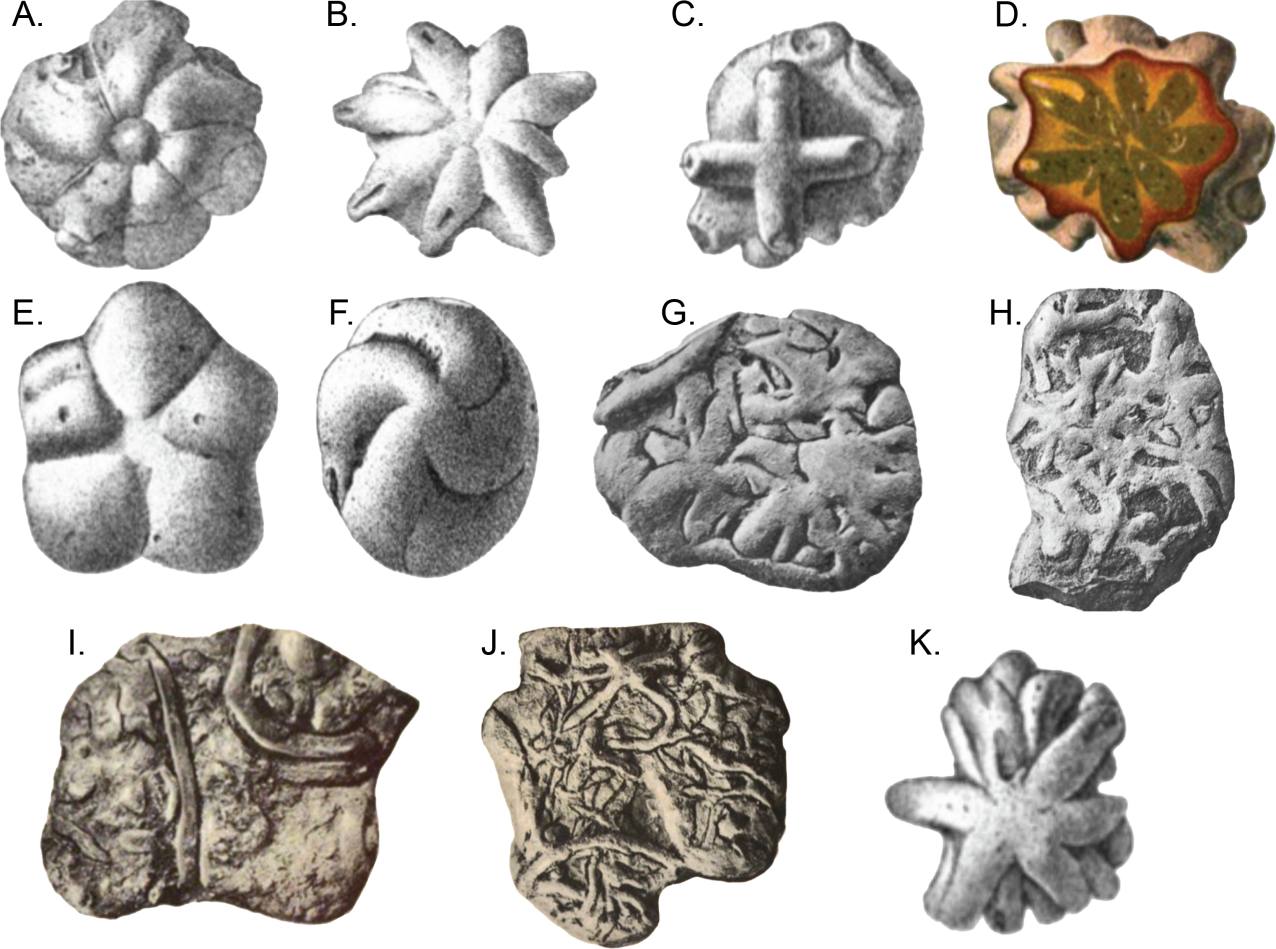
*Brooksella* and *Brooksella*-like fossils synonymized by Ciampaglio et al. (2006) and additional *Brooksella*-like fossils depicted by Walcott from the Conasauga Formation. (A–D) *Brooksella alternata*; (E–H) *Laotira cambria*; (I) annelid trace fossils (*Planolites* sp.); (J) annelid burrows with *Laotira cambria*; and (K) *Brooksella confusa*. Figures from (Walcott, 1898): (A) plate I, Fig. 1; (B), plate I, Fig. 6; (C) plate II, Fig. 8A; (D), plate IV, Fig. 5; (E) plate V, Fig. 7; (F) plate V, Fig. 6; (G) plate XIII, Fig. 2; (H) plate XIV, Fig. 2; (I) plate XV, Fig. 1; (J) plate XV, Fig. 5; (K) plate III, Fig. 12.

The question regarding *Brooksella’s* placement as a sponge, and more specifically, a hexactinellid sponge that could have produced enough biogenic silica to preserve an entire middle Cambrian Lagerstätte, might not yet be settled. Ciampaglio et al. (2006) observed that the external surfaces and cross sections of *Brooksella* had white *Protospongia*-type spicules, four-rayed spicules of siliceous composition. Even so, others suggest the presence of such hexactine spicules are not sufficiently diagnostic for hexactinellids (*e.g.*, Botting & Muir, 2018) and *Protospongia* had calcitic or biminerallic, but not necessarily siliceous spicules (*e.g.*, Page, Butterfield & Harvey, 2009). This calls into question what Walcott keenly observed, however: despite the hundreds of specimens he examined, he found no spicules in thin-section, and, based on a compression of *Brooksella* in shale, he favored a jellyfish fossil over a sponge identity (Walcott, 1898, his p. 21–22). Ciampaglio et al. (2006) also noted ostia (incurrent pores), a central canal (spongocoel), radial canals in each of the numerous lobes, and openings at the tips of the lobes into these canals (refer to their Fig. 3). They also inferred that the concave side of *Brooksella* with the central depression was the top of the specimen, contrary to Walcott’s medusoid interpretation (Ciampaglio et al., 2006, their p. 264). Lastly, Ciampaglio et al. (2006) synonymized three species of Walcott’s *Brooksella* and *Brooksella*-like fossils—*Brooksella alternata*, *Brooksella confusa* and *Laotira cambria*—all of which have variable morphologies and some were associated with annelid traces or trackways (Fig. 1; Walcott, 1898).

**Figure 2:**
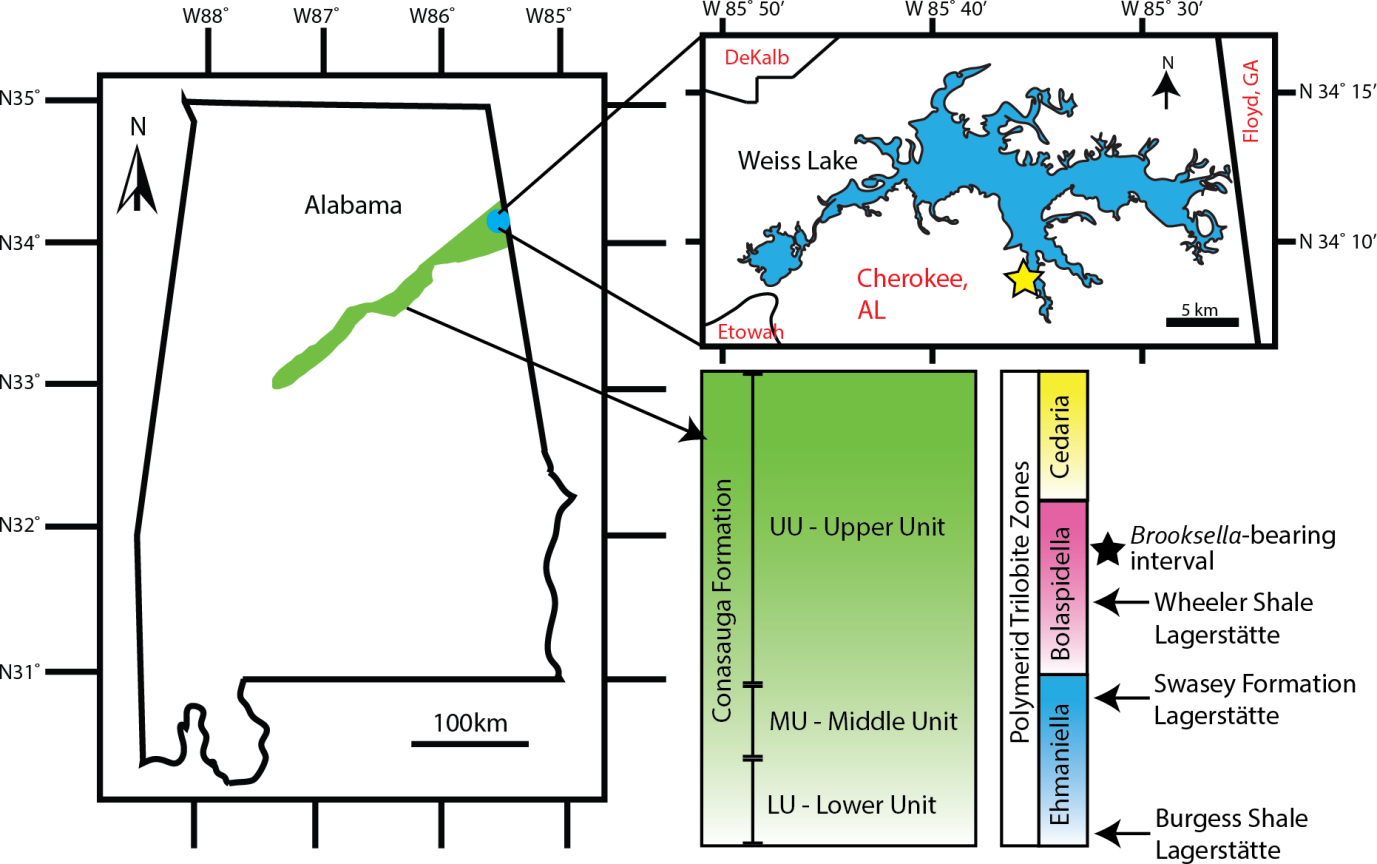
*Brooksella* and concretion field locality in northeastern Alabama, USA. Green area indicates the Conasauga Formation and is linked to the stratigraphic position of *Brooksella* (Map data ©2022 Google; biostratigraphic column adapted from Schwimmer & Montante, 2007). Inset shows Weiss Lake where *Brooksella alternata* were collected, indicated with a star ∼34°08′20″N, 85°35′56″W.

**Figure 3:**
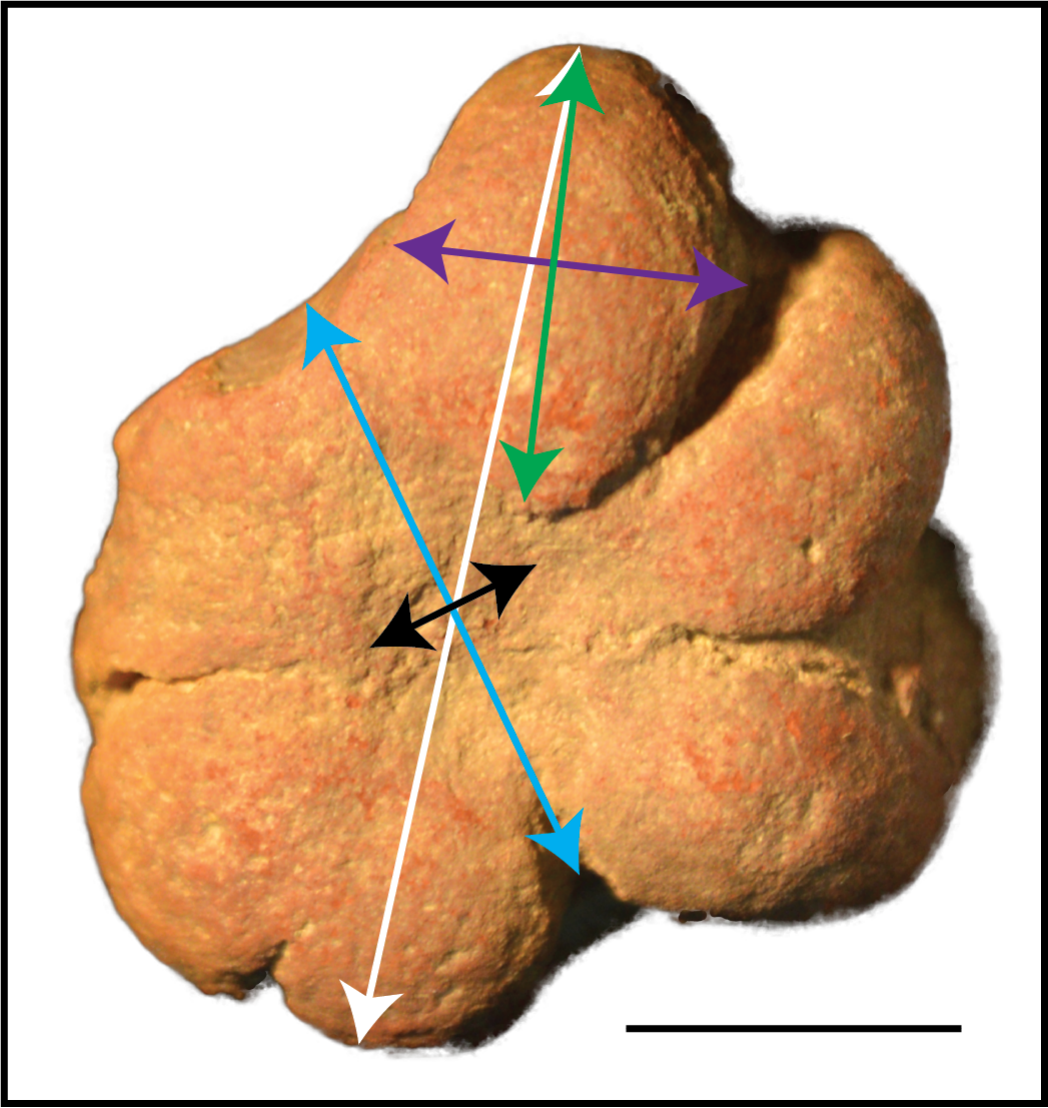
Measurements used to examine *Brooksella* size and morphology. The longest axis of *Brooksella* (maximum diameter; white line); shortest axis (minimum diameter; blue line); maximum lobe length from base to tip (green line); maximum lobe width (purple line); and central depression diameter (black line). Scale bar = one cm; sample UGA WSL2.AL5 depicted.

To resolve whether *Brooksella* is a fossil hexactinellid sponge—which would be critical for producing the biogenic silica needed to preserve the Lagerstätte—or a trace or pseudofossil, the following must be addressed: (1) abundance of *Brooksella* in the field; (2) its orientation within the sedimentary beds; (3) an evaluation of its putative sponge-like characteristics, such as possessing ostia, spongocoel, radial canals in the lobes, *Protospongia*-like spicules on the external surface, spicules on the surface of cross sections, and growth characteristics consistent with known fossil hexactinellids; and (4) whether it has trace fossil characteristics, such as back filling spreiten and evidence of probing. Herein, we reassess whether *Brooksella* is a hexactinellid sponge or trace fossil. We also considered whether *Brooksella* is similar in size and composition to co-occurring concretions, as it may also be a pseudofossil.

### Taxonomic background

Most *Brooksella* and *Brooksella-*like fossils were synonymized by Ciampaglio et al. (2006) as one species, *Brooksella alternata.* Based on superficial appearance, Ciampaglio et al. (2006) synonymized *Laotria cambria* and *Brooksella confusa* (Walcott, 1896; Walcott, 1898) with *Brooksella alternata,* although, *B. alternata, B. confusa,* and *L. cambria* have different external characteristics (Table S1). Ciampaglio et al. (2006) also assigned ?*Brooksella* material from the Spence Shale of Utah (Willoughby & Robison, 1979; Robison, 1991) to possibly *Brooksella alternata*, extending the range of *Brooksella* into the older *Glossopleura* Zone in the Wuliuan stage of the lower middle Cambrian. Additionally, Caster (1942) identified a specimen of *Laotira cambria* from the Cambrian Furogian Series of Wyoming that was later reassigned to *Brooksella cambria* (Harrington & Moore, 1956), and is tentatively considered *B. alternata* by Ciampaglio et al. (2006). *Brooksella silurica* (Von Heune, 1904) includes an Ordovician specimen from Sweden, expanding both *Brooksella*’s geographical range beyond North America and temporal range out of the Cambrian Period (Harrington & Moore, 1956). *Brooksella canyonensis* (Bassler, 1941), found in the Neoproterozoic Grand Canyon Series of Arizona, was reassigned to the trace fossil ?*Asterosoma canyonensis* (Glaessner, 1969; see also Häntzschel, 1970; Kauffman & Fürsich, 1983), but the assignment as a trace fossil is questioned by Ciampaglio et al. (2006). Ediacaran-aged *Brooksella* sp. material from the Nasep Member of the Urusis Formation in the Schwarzrand Subgroup of Namibia was interpreted as a probing trace fossil (Crimes & Germs, 1982). Based on these reports, the most common alternative identity for *Brooksella* is that of a probing, radial trace fossil, like *Dactyloidites*, but the trace fossil attribution for *Brooksella* needs reassessment (Muñoz, Mángano & Buatois, 2019). Thus, in addition to reevaluating the hexactinellid interpretation, we are also examining *Brooksella* for trace fossil characteristics, such as back-filled spreiten, central shafts, and sedimentary relationships like probing structures or movement in relation to the sediment (after Muñoz, Mángano & Buatois, 2019). Herein, we refer to *Brooksella alternata* and its related synonymized species as *Brooksella*.

### Geological setting

The middle Cambrian Conasauga Formation is a predominantly grey shale unit with limestone interbeds that crops out in several southeastern US states: Alabama, Georgia, Tennessee, and Virginia (Palmer & Holland, 1971; Hasson & Haase, 1988). Formal subdivision of the formation varies by state. In Tennessee, the Conasauga is treated as a group and is divided into six formations, each mainly shale or limestone in composition (Hasson & Haase, 1988). Comparatively, in Georgia and Alabama, division of the Conasauga Formation either follows Tennessee’s geologic format (Butts & Gildersleeve, 1948; McLemore & Hurst, 1970), or it is a formation informally divided into lower, middle, and upper portions (Cressler, 1970; Chowns, 1977).

The Coosa Valley, northeastern Alabama, is the source of all *Brooksella* and concretions in our study and is the primary source of *Brooksella* for Walcott’s (1896; 1898) studies. Part of the Appalachian Valley and Ridge Province (Butts, 1926; Cressler, 1970; Thomas, 1985; Osborne, Thomas & Astini, 2000), the Coosa Valley localities are topographically low, with substantial vegetation cover, extensive faulting, and are mostly submerged by the Weiss Lake reservoir, thus, limiting fine stratigraphic correlation among localities (see also Ciampaglio et al., 2006). Chert nodules weather out of several shaley stratigraphic units. The chert and *Brooksella*-bearing layers are found at times associated with lenticular carbonate beefs and polymerid trilobites of the *Bolaspidella* Zone (Schwimmer, 1989), which provides constraint to the Drumian Stage of the middle Cambrian (504.5 to 500.5 mya; Cohen et al. (2018)). Carbonate nodules also weather out from stratigraphically lower shale units, but not in the units where we collected *Brooksella*.

The fossils of the Conasauga Formation are comparable in generic richness to the Wheeler and Spence Shales of Utah (Schwimmer, 2000), though the degree and quality of preservation is much poorer than the Wheeler or Spence Shales. Facies interpretations suggest likely deposition in a restricted paleoenvironment (Robison, 1991) that is generally shallower than the Wheeler Shale and other Burgess Shale-type facies (Schwimmer & Montante, 2007).

The Conasauga Formation preserves fossils in two forms: flattened organic or ferrous impressions on shales and 3-D silicified materials on or within chert concretions (Schwimmer & Montante, 2007). The 3-D preservation of some fossils has led to the description of the Coosa Valley localities as Konservat-Lagerstätten (Schwimmer & Montante, 2007). Soft-bodied organisms and structures preserved in the Conasauga Formation include red algae, green algae, priapulids, and nektaspids (Schwimmer & Montante, 2007).

## Material and Methods

### Sample collections

*Brooksella* samples (*n* = 77) come from three sources: existing University of Georgia (UGA) collections from the second author (*n* = 29), samples donated by Dr. Donald Champagne (*n* = 27), and by additional field collections from the Coosa Valley for this research (*n* = 21). These samples are currently held at the UGA Department of Geology but will be reposited with the Smithsonian National Museum of Natural History. No permits were required for the described study, which complied with all relevant regulations for the State of Alabama. All samples were collected along the banks of Weiss Lake, Cherokee County, Alabama (Fig. 2). However, collection is limited to the winter months when the Weiss Lake reservoir water level is lowest, and the banks are exposed. *In situ Brooksella* and concretions were collected with their locations and positions noted along six transects arrayed along exposed in place (not overturned) shale beds that parallel the lake shore. Additional *Brooksella* and concretions that were not *in situ* were collected as float below the transects. To compare to the *Brooksella*, we additionally examined siliceous concretions from the same localities (*n* = 98 siliceous concretions from existing UGA collections and from additional field collection) and *n* = 1 carbonate concretion from another locality. Additionally, images of figured specimens of *B. alternata* (*n* = 33), *B. confusa* (*n* = 3), and *L. cambria* (*n* = 58) from Walcott (1898) were examined to collect size data, orientation of lobes, and number of lobes to compare to our samples; according to Walcott (1898), all images were life size.

### *Brooksella* and concretion surficial analysis

The surfaces of *Brooksella* and concretions were observed *via* optical microscopy before and after cleaning the samples, which had clay, lichen and algae on them. For *Brooksella* and concretions, we noted the presence or absence of the following surficial features attributed to sponges by Ciampaglio et al. (2006): Central depression (osculum) and small crater-like pores (ostia) as recorded in Table S2.

To quantify the size of *Brooksella* and concretions, digital calipers (accuracy ±0.03 mm) were used to measure the minimum diameter (shortest axis) and maximum diameter (longest axis) (Fig. 3; Tables S2 and S3). As a proxy for general size, we used both maximum and minimum diameter and geometric mean of the maximum and minimum diameter (square root of their product) for statistical applications.

Because lobes are the main diagnostic character of *Brooksella* and purportedly house the internal radial canals of the sponge, we first noted where the lobes occurred, either the top or bottom surfaces or both surfaces, for each specimen. We also counted the number of lobes per surface and measured the largest lobe length and width with digital calipers. The lobe length and width measurements were converted to geometric means to compare to the size of *Brooksella.*

Lastly, images of *B. alternata*, *B. confusa* and *L. cambria* from Walcott (1898) were measured with digital calipers for maximum and minimum diameter. For analysis, the data from the three species were pooled as Walcott’s *Brooksella* to compare to our *Brooksella* and concretions. Further, the number of lobes were counted and if possible, their occurrence on one or both surfaces was also noted. Central depressions were not always depicted and therefore not measured; lobe width or length were also not measured from these images as it was often not possible to determine their dimensions on all specimens. These *Brooksella* are referred to as “Walcott’s *Brooksella*” to distinguish them from our own collections.

To compare the maximum and minimum diameters among our *Brooksella,* concretions, and Walcott’s *Brooksella*, the measurements were converted to a geometric mean and grand geometric mean and plotted with their 95% confidence intervals (95% CIs were from a one-sample *t*-test for each type; R Core Team, 2021). The relationship between maximum and minimum diameter (without geometric mean) among *Brooksella*, concretions and Walcott’s *Brooksella*, was examined using Model II standard major axis regressions (SMA) with 95% CIs for the slope. These were calculated and plotted in R (Legendre, 2018; R Core Team, 2021; package lmodel2). Model II regressions were used because the two variables measured were not controlled by the researcher unlike in a Model I regression (Legendre, 2018). The null hypothesis for this test was that there was no difference in the relationship between maximum and minimum diameter between all three sample types.

Top lobe frequency of occurrence was examined by size class for our *Brooksella* and Walcott’s *Brooksella* to determine which size class or classes the lobes most commonly occur. A generalized linear model (GLM) with quasiPoisson for over-dispersed lobe count data was used to determine if the number of lobes increase as the size of *Brooksella* increase for both our samples and those of Walcott’s *Brooksella* (R Core Team, 2021). A Model II SMA regression was used to examine the strength of the relationship between the geometric mean size of the largest lobe and the geometric mean size in our *Brooksella* and Walcott’s *Brooksella*; correlation coefficients were determined using the cor.test function in R (Legendre, 2018; package lmodel2; R Core Team, 2021).

### *Brooksella* and concretion internal structure

Internal analysis of *Brooksella* and concretions was conducted using three methods. First, we cross-sectioned eleven *Brooksella* and two silica and carbonate concretions to try to locate the central cavity (spongocoel), radial canals and white spicules that Ciampaglio et al. (2006) reported from the surface cut area. Second, eleven *Brooksella*, two siliceous and one carbonate concretion were polished and made into petrogaphic thin sections to examine their composition and to also determine whether a spongocoel, radial canals, ostial chambers, and an external thin spicular wall were present. The thin sections were prepared by Vancouver Petrographics Ltd, British Columbia, Canada. Lastly, to visualize any internal features including spongocoel, radial canals, or trace fossil characteristics, *Brooksella* (*n* = 21) and concretions (*n* = 6) were scanned with the UGA College of Veterinary Medicine’s Computed Tomography (CT) scanner (a Siemens Sensation 64 slice unit; scans were collected under 120 kVp, a tube current of 190 mA, slice thickness of 0.6 mm, and convolution kernel setting of B80s for sharp/bone kernel). Additionally, two *Brooksella* and two silica concretions from this set were also scanned at a higher resolution using a Zeiss Xradia 510 Versa *μ*CT microscope at the University of Missouri X-ray Microanalysis Core Facility. Micro-CT scans were collected at 80 kV, 7 W, 2001 projections, 2–7 s of exposure, optical magnification of 0.4X, 360 degrees of rotation, a Zeiss LE6 filter, and a pixel size of between 50.3–58.4 µm. The CT and *μ*CT image stacks are available as supplemental data on morphosource, as Project ID: 000436718, *Brooksella* and silica concretions.

### *Brooksella* and concretion compositional analysis

To determine and compare bulk compositions between *Brooksella* and concretions, portions of two *Brooksella* and two siliceous concretions were powdered *via* ball mill and scanned with a Bruker D8 Advance X-ray Powder Diffractometer (XRD) at UGA. To examine the elemental composition of specific internal structures, petrographic thin sections from *Brooksella* and siliceous concretions (*n* = 2 each) were carbon coated and analyzed using a JEOL 8600 electron microprobe (EPMA) at the UGA Department of Geology. Backscattered electron images and energy dispersive X-ray (EDS) maps were processed with the Bruker Quantax analysis system.

## Results

### Field abundance and orientation of *Brooksella* and concretions in the Conasauga Shale

*Brooksella* were rare in the shale outcrops at Weiss Lake. Field transects of *in situ Brooksella* only occurred with a frequency of 0.10 for all transects combined (Table 1). Many more *Brooksella* and concretions were found as float located below the transects, but float *Brooksella* occurred at a lower frequency than that of collected float concretions (Table 1).

**Table 1:** Field occurrence of *in situ* and float *Brooksella* and concretions from six transects totaling 75.2 m in length.

***In situ Brooksella*/frequency**	***In situ* concretions/frequency**	***Brooksella* float/frequency**	**Concretion float/frequency**
2/0.10	18/0.90	13/0.25	39/0.75
Per meter	Per meter	Per meter	Per Meter
0.02	0.24	0.17	0.52

**Figure 4:**
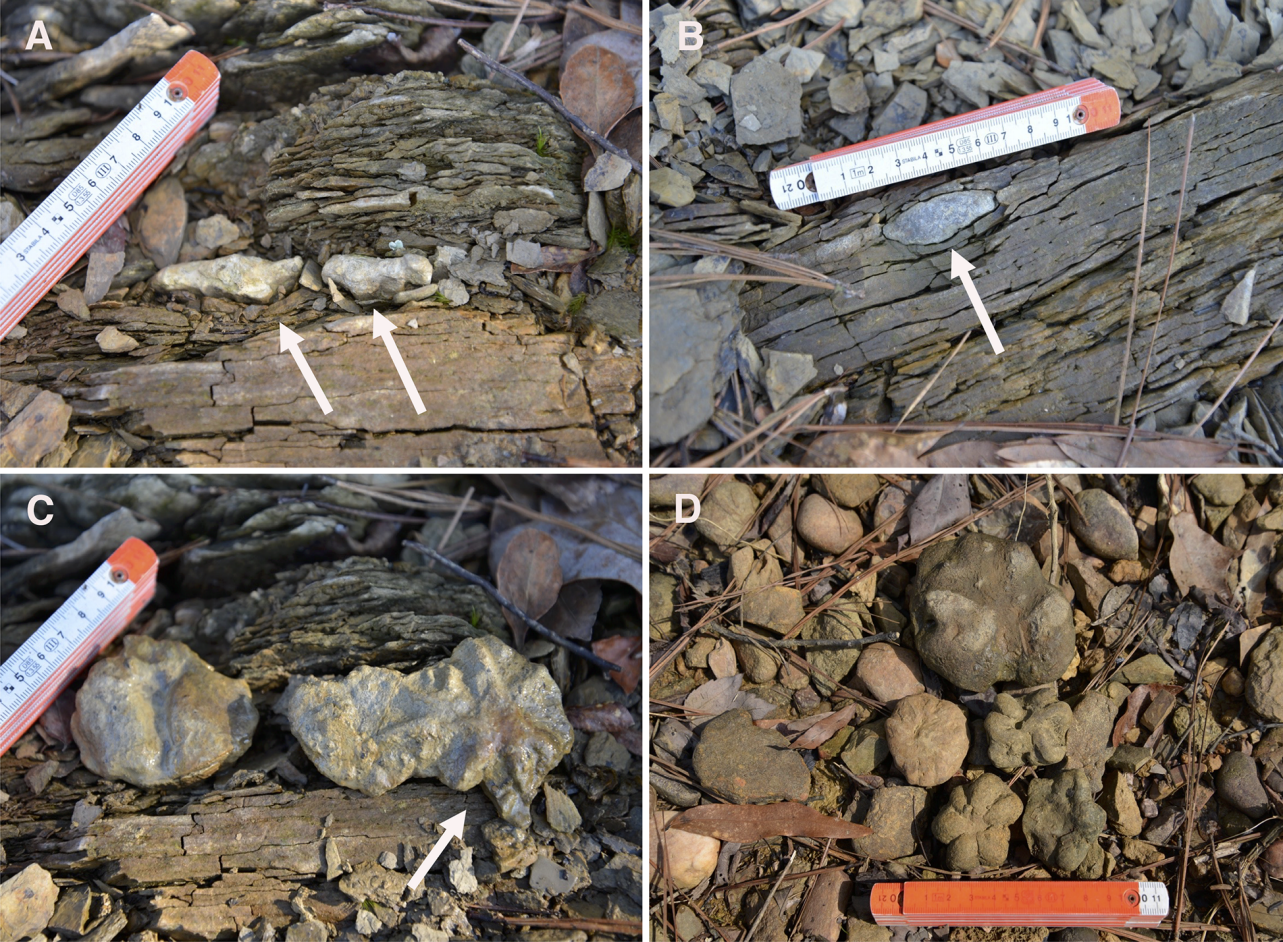
*In situ Brooksella* and concretions from Weiss Lake locality. (A) Sediment layers below specimen are deformed around *Brooksella* (left arrow); lobes of *Brooksella* are oriented downward into sediment (right arrow). (B) *In situ* concretion in shale with its most convex side downward (arrow); it also deforms the shale layers around it. (C) *Brooksella* depicted in A but now oriented upward (arrow). (D) Float *Brooksella* and concretions. Centimeter ruler for scale; *Brooksella* samples: UGA 1,2, 8, and 5.

*In situ Brooksella* were oriented in the shale with the stellate lobes on the concave surface facing downward into the sediment; *Brooksella* also appeared to deform the shale laminae (Fig. 4A). Concretions also had their more concave side oriented downward into the sediment and they also deformed the shale laminae around them (Fig. 4B). The *Brooksella* removed from the shale depicted in Fig. 4A appeared twinned (Fig. 4C). *Brooksella* and concretions co-occurred as siliceous cobbles on the shoreline of Weiss Lake in our locality (Fig. 4D).

**Figure 5:**
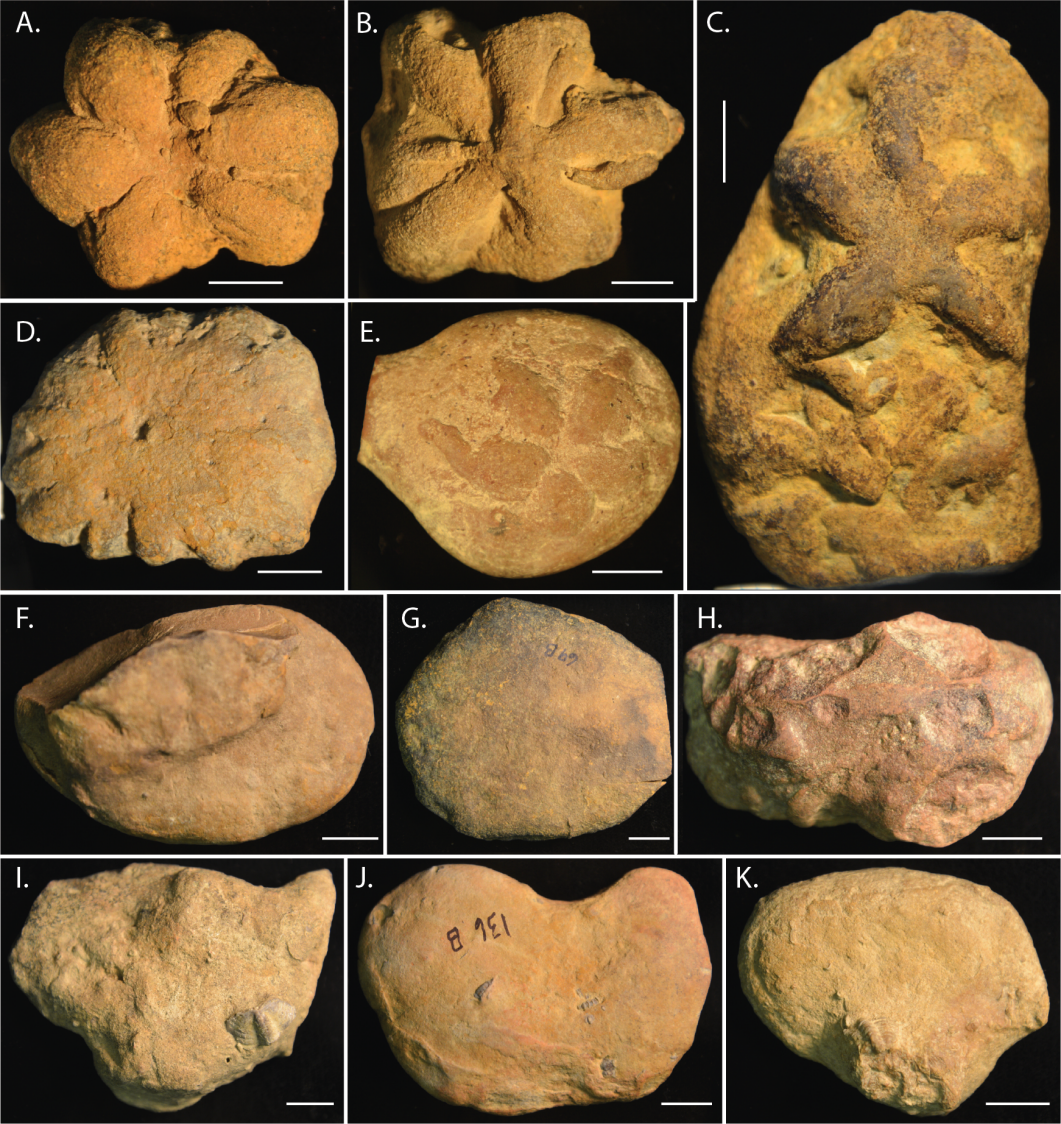
Morphological diversity in *Brooksella alternata* and concretions from Weiss Lake locality. *Brooksella* shapes are variable: typical *Brooksella* have approximately six lobes (A, B); twinned *Brooksella* can also occur (C); others can have multiple indistinct lobes (D) or lobes that are completely embedded in a concretion (E). Concretions (F–K) also vary in shape, but are mostly round to oblong and many have fossils fragments or whole trilobites embedded in them. Scale bars = one cm. *Brooksella* figured: (A) UGA 1; (B) UGA WSL2.AL2; (C) UGA WSL2.AL16; (D) UGA WSL2.AL4; (E) UGA LSV1.AL2; concretions figured: (F) UGA 40; (G) UGA 69; (H) UGA 25; (I) UGA 73; (J) UGA 136; (K) UGA 22.

**Figure 6:**
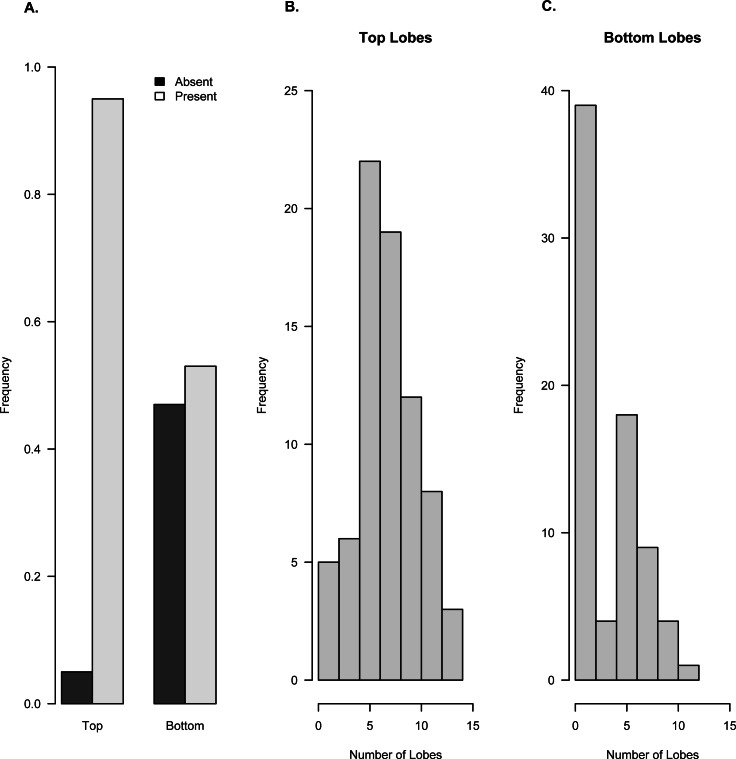
Frequency of occurrence of lobes on top and bottom surfaces of *Brooksella* (*n* = 71). Based on field orientation, the top surface (with top lobes) faces downward into the sediment and the bottom surface (with bottom lobes) faces upward. (A) Presence/absence frequency of lobes on top and bottom surfaces. Histograms of the number of lobes on the top surface (B) and bottom surface (C).

**Figure 7:**
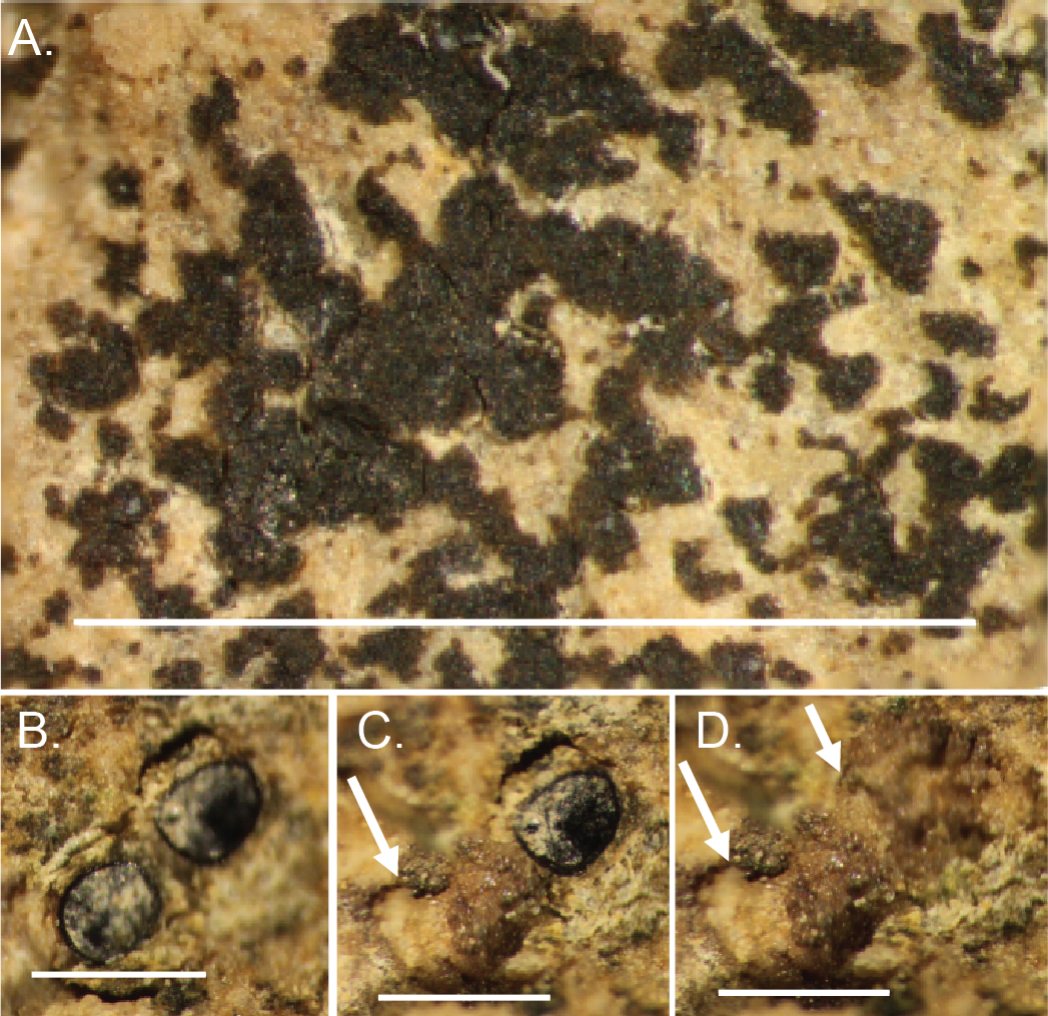
Lichen attach to and bioerode the surface of *Brooksella*. (A) *In situ* lichen; (B) close up of lichens; (C) same image as (B), but lichen was removed, revealing a bioerosion pit (arrow); (D), surface view of bioerosion pits (arrows) made by lichens on *Brooksella* surface after lichen were removed. Scale bars: (A) = one cm; (B–D) = one mm.

### External morphology of *Brooksella* and concretions

The external morphology of *Brooksella* was variable in both the number of lobes and whether the central depression was present or not. A typical *Brooksella* had well-defined lobes and a central depression (Fig. 5A), which is referred to as the top surface of *Brooksella* by Ciampaglio et al. (2006) and the bottom surface of a jellyfish by Walcott (1898); however, we refer to it as the top surface to be consistent with Ciampaglio et al. (2006) although this side is facing downward into the sediment. Only 38% of *Brooksella* had a central depression, while some (*n* = 5, or 6.5% of all specimens) had a central protuberance (Fig. 5B). The remaining 55.5% had no discernable central depression or protuberance (Fig. 5C). While *Brooksella* are usually depicted as having lobes extending to the margins of the specimen (Figs. 5A–5B), they do not always have this feature (Fig. 5C–5E). Some specimens (*n* = 5) display multiple individual sets of lobes, although the second set of lobes is usually indistinct (Fig. 5C). We did not observe spicules on the external surfaces of *Brooksella*. Concretions from the Conasauga also display variable morphology (Figs. 5F–5K); some have visible trilobites or trilobite fragments on their surfaces (Fig. 5I).

Lobes are more common on the top surface of *Brooksella* that is oriented downward into the sediment and least common on the bottom surface which is oriented upward in the sediment (Fig. 6). Ninety-four percent of *Brooksella* have top surface lobes while 55% have bottom surface lobes (Fig. 6A), and half of the *Brooksella* have lobes on both sides (*n* = 35, 0.49 frequency). Five and six lobes are the most common on top surfaces, ranging from a few with no lobes to one specimen with 15 lobes (Fig. 6B). Having no lobes was most common on the bottom surface, followed by five lobes, with a maximum number of 12 lobes (Fig. 6C). Importantly, none of the lobes had openings at their ends that would indicate a radial canal opening.

### Pits on the surface of *Brooksella*

The surfaces of *Brooksella* are host to lichen colonies, which can be abundant (Fig. 7A). The lichen can be peeled off the surface, revealing small round indentations approximately 0.05 mm in diameter (Figs. 7B–7D). Concretion surfaces had similar lichen and algal colonies.

### Size relationships of *Brooksella* and concretions

### Overall size

**Figure 8:**
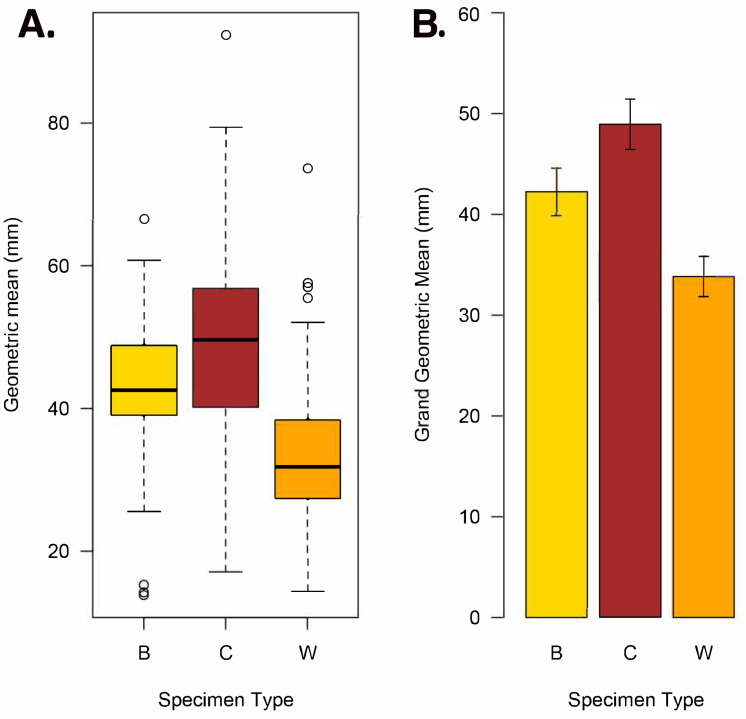
Geometric mean (square root of maximum diameter × minimum diameter) and grand geometric mean size comparison among *Brooksella*, concretions, and Walcott’s *Brooksella*. (A) Boxplots of geometric mean. (B) Barplot of grand geometric mean with 95% CI error bars. Specimen type key: B = *Brooksella*, C = concretions, and W = Walcott’s figured *Brooksella*.

Based on the geometric mean, concretions were more variable in size and generally larger than either *Brooksella* or Walcott’s *Brooksella* (Fig. 8A). Generally, the size distribution of *Brooksella* overlaps with the smaller sizes of the concretions (*i.e.,* below the median for concretions). However, our *Brooksella* are larger than Walcott’s *Brooksella*. Concretions had a slightly larger grand geometric mean size (48.92 mm) than *Brooksella* (42.22 mm), but both were much larger than the grand geometric mean for Walcott’s *Brooksella* (33.82 mm; Fig. 8B). There was a significant difference among all the specimen types for the grand geometric mean, as none of the 95% CIs overlapped (Fig. 8B).

**Figure 9:**
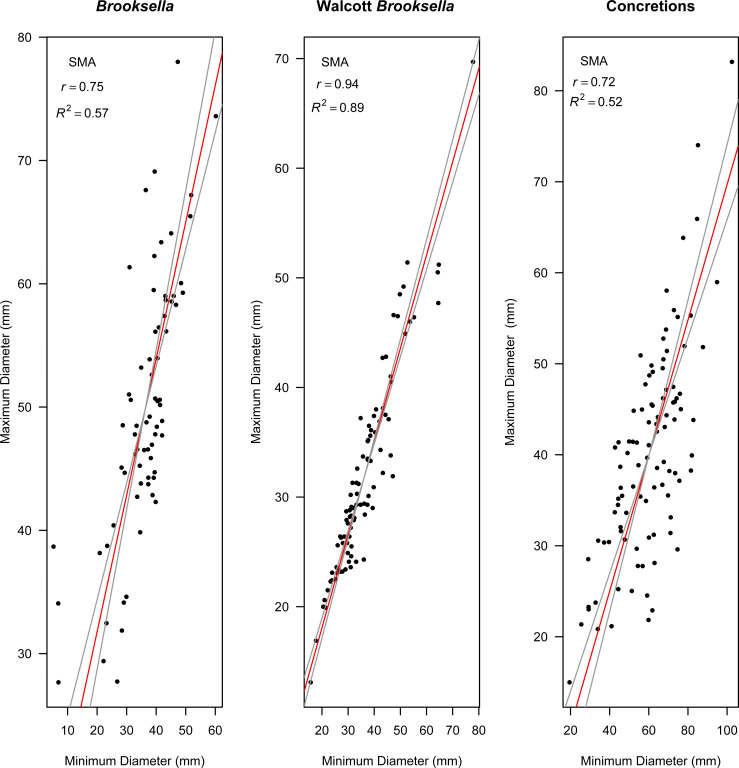
Model II standard major axis (SMA) regressions between maximum and minimum diameter for *Brooksella*, Walcott’s *Brooksella*, and concretions. 95% CIs for the slope are depicted as grey lines around the slope (red line).

Model II regressions indicate that maximum and minimum diameter among the specimen types had positive relationships and the correlation tests indicated that they were moderately to well correlated (Figs. 9A–9C). Walcott’s figured samples were highly correlated, and the Model II regression slope explained 89% of the data (Fig. 9B). However, maximum and minimum diameters were only moderately correlated for our *Brooksella* and concretions; the regression slopes only explained half of the data (57% and 52%, respectively; Figs. 9A and 9C).

### Number of top lobes in relation to *Brooksella* size

**Figure 10:**
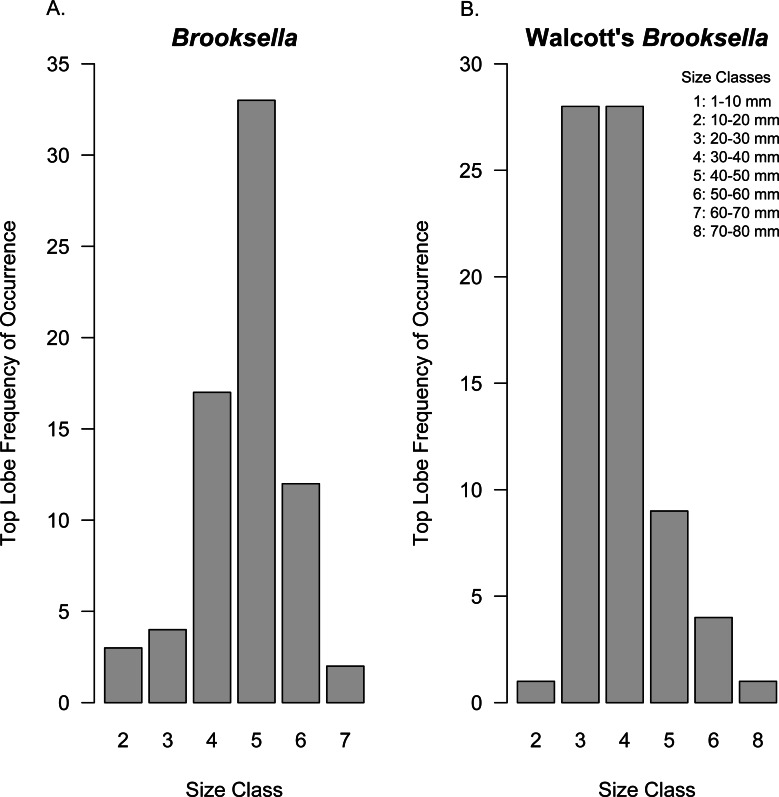
Top lobe frequency of occurrence by size class for *Brooksella* (A) and Walcott’s *Brooksella* (B). Size is based on the geometric mean.

**Figure 11:**
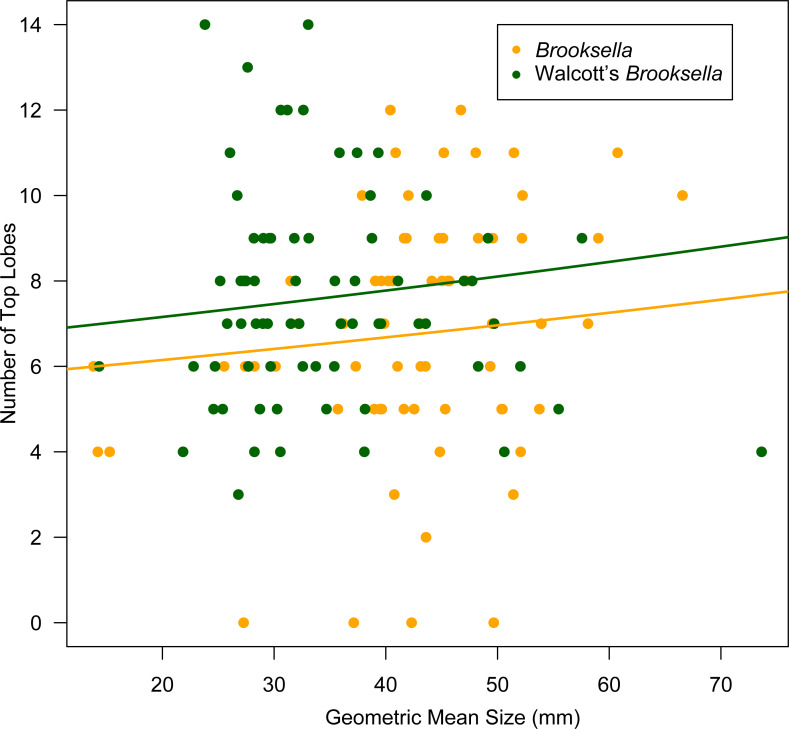
Generalized linear regression between number of top lobes in relation to geometric mean size in *Brooksella* and Walcott’s *Brooksella*.

**Figure 12:**
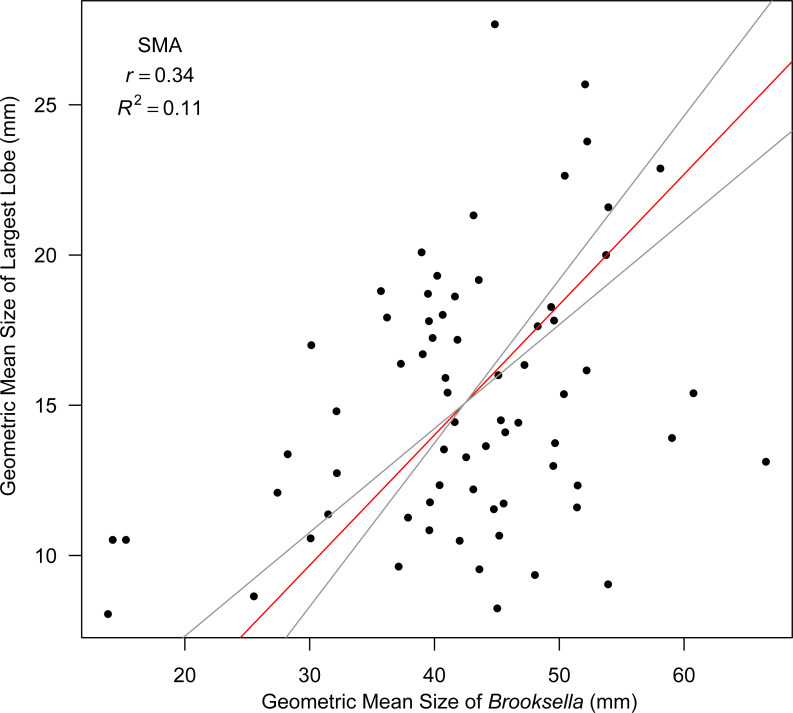
Model II SMA regression for geometric mean size of largest lobe in relation to geometric mean size in *Brooksella*. Slope (red line) is depicted with 95% CIs (grey lines).

Top lobe occurrence in relation to size class based on geometric mean was different between our *Brooksella* and that of Walcott’s (Fig. 10). Top lobes occurred more frequently on *Brooksella* that were 40 to 50 mm in size (size class 5; Fig. 10A), while for Walcott’s *Brooksella*, they occurred more frequently on specimens that were 20 to 40 mm in size (size classes 3 and 4; Fig. 10B). In general, the number of top lobes barely increased with size for both our *Brooksella* and Walcott’s specimens; essentially, it was nearly a flat slope for the generalized linear model regression (Fig. 11). Moreover, although it appears that as *Brooksella* gets larger, its largest lobe also increases in size, the data only accounted for 11% of the slope and the correlation coefficient was extremely low ( *r* = 0.34), indicating that there was no relationship between the largest top lobe size and overall *Brooksella* size (Fig. 12).

### Internal structure and composition of cross-sectioned *Brooksella* and concretions

**Figure 13:**
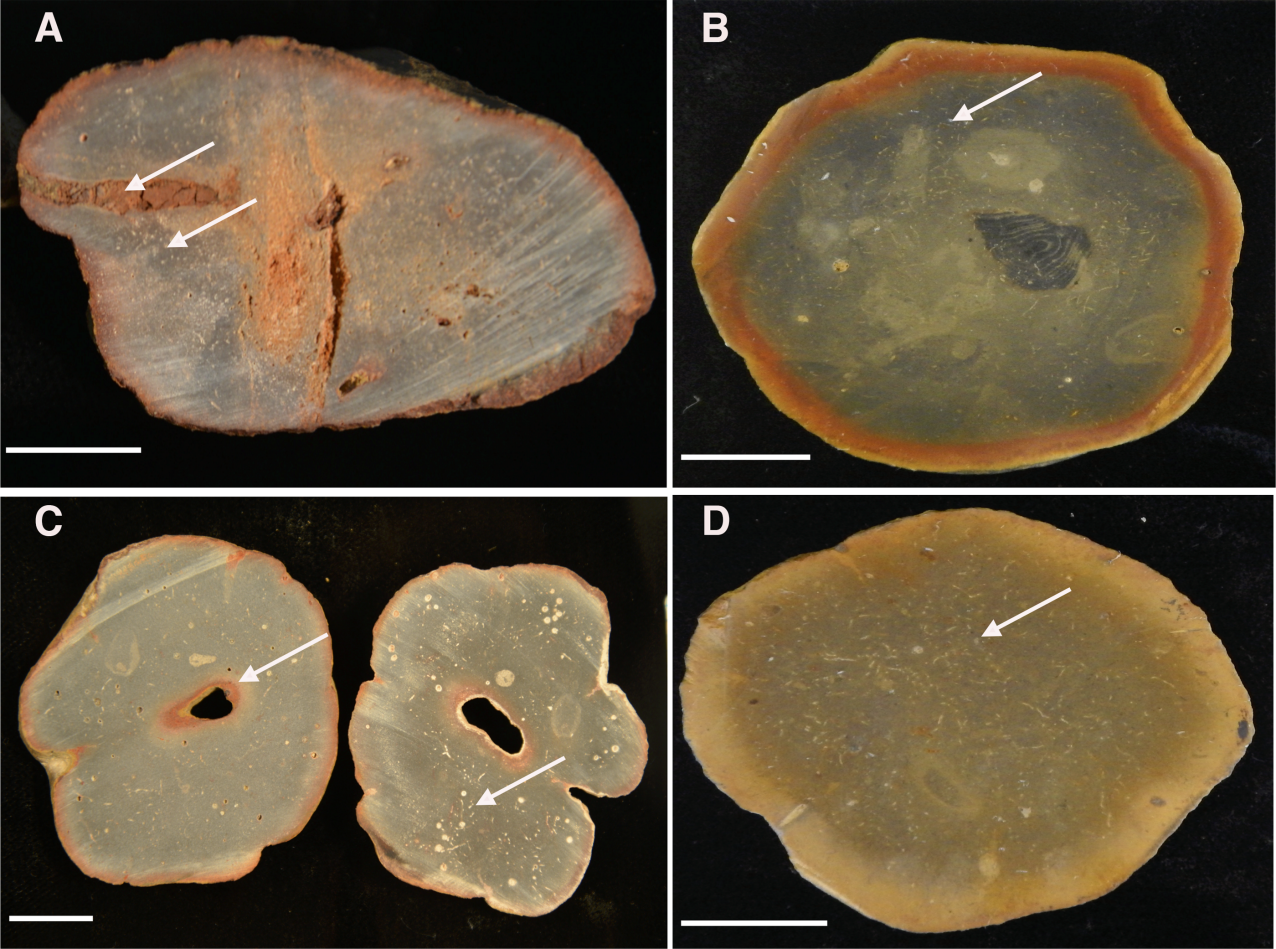
Cross-sectioned concretions (A–B) and *Brooksella* (C–D) showing iron-oxide weathering rind and internal surface structures. (A) Concretion dissected by root bioerosion (upper arrow) and marked by voids (lower arrow) that appear white in photographs; (B) concretion with weathering rind, variable internal coloration that is not concentric in form, and has white-appearing voids (arrow); (C) *Brooksella* that was affected by roots, which formed an oxidized hole in the center (arrow) on left cross-section and internal composition is variable with numerous voids and tubes that appear white in photographs but are not spicules (arrow, right cross section); (D) *Brooksella* with nearly homogenous internal texture, with voids and tubes (arrow). Scale bar: one cm. Figured specimens: (A) UGA 126; (B) UGA 156; (C) UGA WSL2.AL21; (D) UGA WSL2.AL1.

Cross-sectioned *Brooksella* and concretions have oxidized weathering rinds (∼2 mm thick); they also have similar internal structures, similar textural variability, and occasional root bioerosion (Fig. 13). Internal color is variable, including grey (Fig. 13A), dark grey and black (Fig. 13B), and lighter grey-brown (Figs. 13C–13D). There were no typical internal concentric bands of differing color for either specimen type and no indication of encapsulating sediment laminations from the surrounding shale.

Sponge-like characters are not evident for either *Brooksella* or concretions on the cross-sectioned sample surfaces. Rather, both *Brooksella* and concretions have what appear at first to be white spots on the surface of the cross sections, but upon closer inspection under a microscope, these are round voids and tube-like structures (Figs. 13A–13D) and were not white spicules. None of the *Brooksella* or concretions have visible hexactinellid sponge-spicule framework near the outer wall, as would be indicative of protospongiids. Importantly, none of the concretions (Figs. 13A–13B) or *Brooksella* (Figs. 13C–13D) have what could be defined as an internal spongocoel, nor do they have radial or lateral canals. Additionally, there are no radiating spreiten.

**Figure 14:**
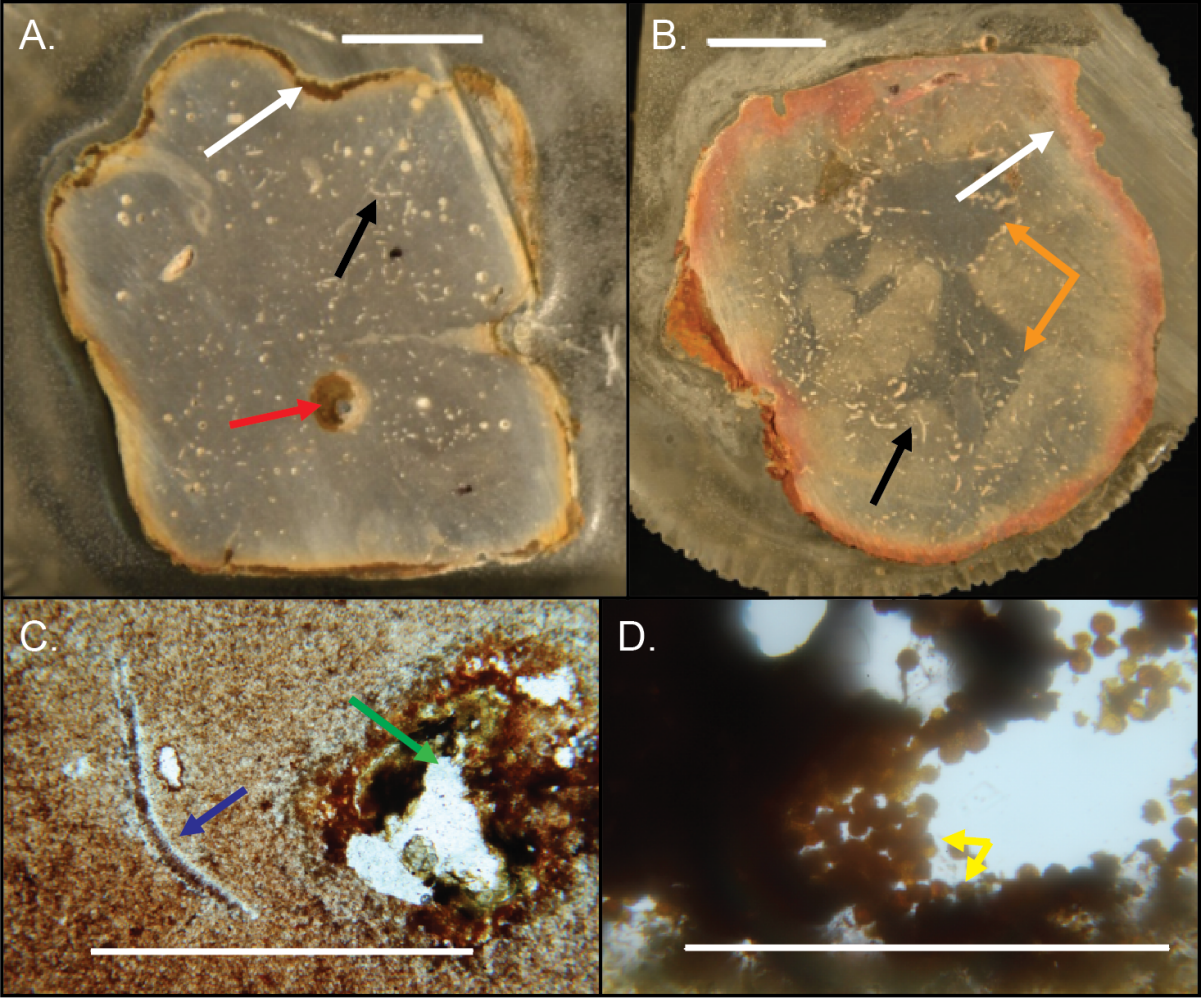
Internal structures in cross-sectioned *Brooksella* (A) and a concretion (B) and petrographic thin sections (C–D). (A) *Brooksella* with weathering rind (white arrow), a large root trace (red arrow) and a curved structure, which is a trilobite fragment (black arrow). (B) Concretion with weathering rind (white arrow), trilobite fragments (black arrow) and dark grey center portion which has a variable shape (orange arrows). (C) Trilobite fragment in *Brooksella* thin section (blue arrow) and diagenetic void (green arrow). (D) thin section of tube within weathering rind of *Brooksella* with framboidal pyrite lining (yellow arrows). Scale bars: (A–B) one cm; (C) one mm; (D) 0.2 mm. Figured specimens: (A) UGA 2; (B) UGA 27; (C) UGA 54; (D) UGA WSL2.AL1.

The small voids and tubes ranged from spherical to irregular in shape and can be either unlined, lined, or partly filled with red and yellow iron oxides and clays (Figs. 14A–14C). Framboidal pyrite is present in some voids (Fig. 14D). There are also curved structures (Figs. 14A–14C), which were often trilobite exoskeletal fragments rich in Ca, Al, and P or were replaced by patchy silica indistinguishable from the surrounding material; other structures were indeterminate but were not spicular skeletal fragments.

### CT scans of *Brooksella* and concretions

**Figure 15:**
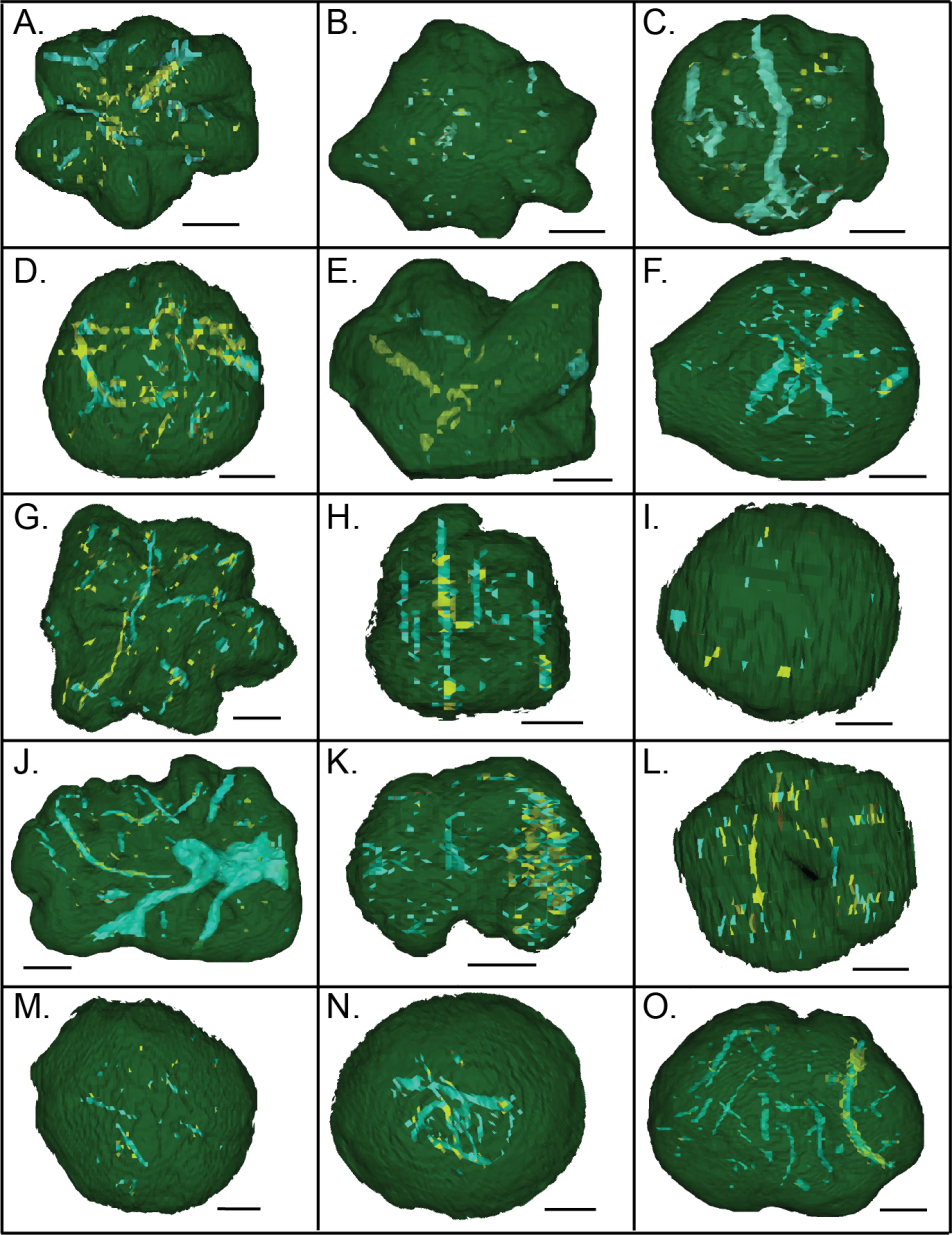
CT scans of *Brooksella* viewed from the top surface (A–L) and concretions (M–O). Green indicates external morphology, blue indicates low density mineral phases and voids, and yellow indicates higher density mineral phases. Scale bar = one cm. (A–L); figured *Brooksella* samples: (A) UGA 1; (B) UGA 3; (C) UGA 6; (D) UGA WSL2.AL1; (E) 55; (F) UGA LSV1.AL2; (G) UGA WSL2.AL2; (H) UGA 98; (I) UGA WSL2.AL12; (J) UGA 17; (K) UGA 155; (L) UGA WSL2.AL21; figured concretion samples: (M) UGA 103; (N) UGA 56; (O) UGA 60.

CT scans revealed that both *Brooksella* and concretions have, in general, internal hollow tubes with random orientations and randomly distributed dense spheres ∼2 mm in diameter (Figs. 15A–15O). Only two of the 12 CT–scanned *Brooksella* had what appeared to be a low-density region in a somewhat stellate shape, but these do not match the location of the lobes (Figs. 15A and 15F), the rest had either cross-sections of low-density regions that appear to be voids or cross-sections of tubes (Figs. 15B, 15I and 15K–15L) or irregular low-density regions, reminiscent of burrows, throughout the matrix (Figs. 15C–15E, 15G and 15J). Some of these tubes are likely mineralized, as represented by the high-density regions within the filled tubes, voids or burrow-like structures (Figs. 15B, 15D–15E, 15G and 15K–15L). Concretions (Fig. 15M–15O) had similar features, with low density burrow-like structures, some of which were filled with high density minerals (Figs. 15N–15O).

**Figure 16:**
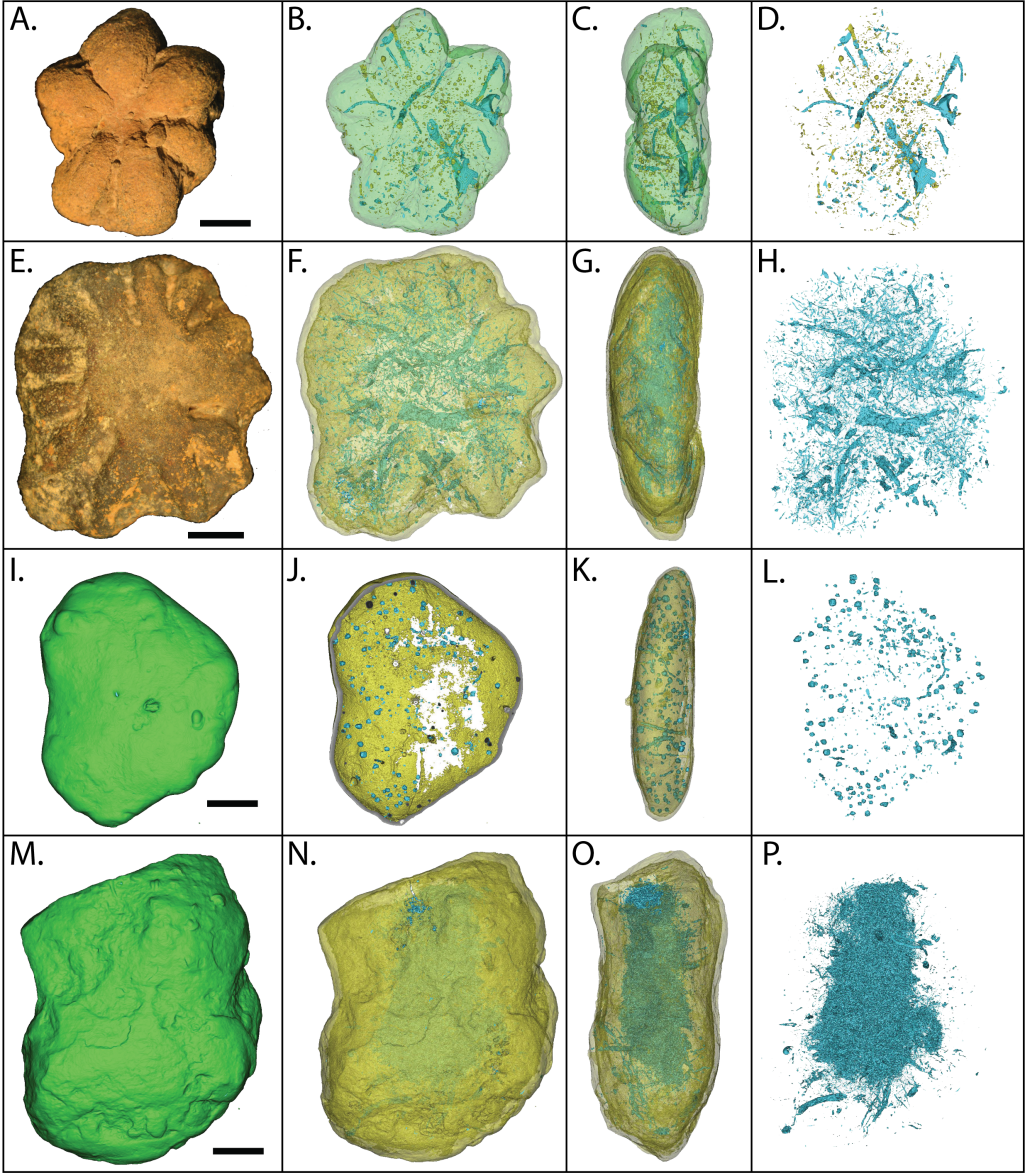
MicroCT reconstructions of the internal structures within two *Brooksella* (A–H) and two concretions (I–P). External (A) and internal (B–D) morphology of a six-lobed *Brooksella;* external (E) and internal (F–H) of a 14-lobed *Brooksella;* and external morphology of two concretions (I, M) and their internal morphology (J–L and N–P, respectively). The first column represents external morphology either in photograph (A, E) or 3-D rendering (I, M); second column represents 3-D reconstruction with the matrix faded to highlight the internal structures (blue represent regions of low density; yellow represents regions of higher density); third column represents 3-D reconstructions of side (profile) view of the specimens; fourth column represents a composite of all the internal features from the serial scans through the specimen. Scale bars = one cm. Figured *Brooksella:* (A–D) UGA 1; E–H, WSL2.AL11. Figured concretions: (I–L), UGA 93; (M–P), UGA 107.

As viewed in high-resolution µCT scans, both *Brooksella* and concretions (Figs. 16A–16P) had extensive internal features defined by mineral phases denser and less dense than the surrounding silica matrix. These features include isolated void-like structures, isolated tubes or burrow-like structures, and fossil fragments. The µCT transmittance values indicate that these structures are represented by low-density mineral phases rather than void space, as compared to the air surrounding the specimen. Several of these tubes have vertical components. Notably, none of the burrow- or tube-like structures occur in the center of the specimen consistent with a spongocoel or central shaft or are in alignment with the lobes.

### Mineral composition of the groundmass and internal structures of *Brooksella* and concretions

X-ray diffractograms of *Brooksella* and siliceous concretions revealed no differences in mineral composition. Both have a composition that is primarily silica with minor calcite, likely occurring as fine cements, interstitial crystals, or biotic hardparts (Fig. S2).

**Figure 17:**
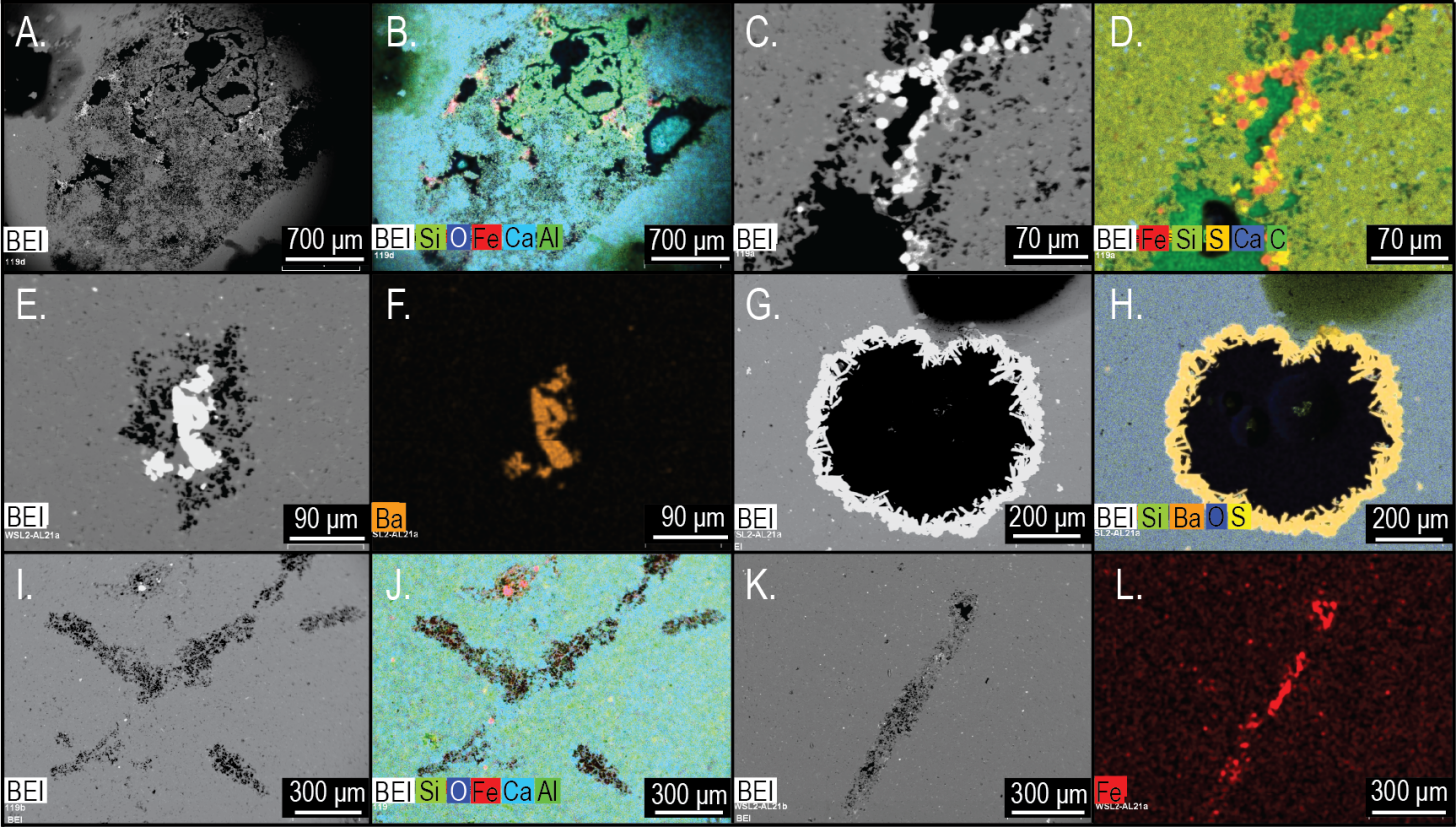
Electron microprobe images of internal structures of two *Brooksella*. Partial void with aluminum and iron oxides (A–B); tubular void that outlets to an external surface lined with framboidal pyrite (C–D); partial void with barite infilling (E–F); void with crystalline barite rim (G–H); cross-shaped void space, lacking skeletal hard parts (I –J); linear structures resembling (I), but with partial iron sulfide composition (K–L). In (A–C, E), and (G–K), void space is black; in (D), void space is dark green. Figured *Brooksella*: (A–D) and (I–J), UGA 119; (E–H) and (K–L), UGA WSL2.AL21.

Electron microprobe analysis of two *Brooksella* specimens corroborated the XRD results, with aluminous silica as the dominant mineralogy but also revealed additional structures and mineral compositions not observed in XRD (Fig. 17). These internal structures include: large voids that are partly filled with iron oxides and aluminosilicates (Figs. 17A–17B); small tubes in the weathering rind lined with framboidal pyrite (Figs. 17C–17D); barite crystals surrounded by microscopic voids (Figs. 17E–17F); round voids lined with barite crystals (Figs. 17G–17H); and cross-shaped structures, perhaps irregular ghosts of stauracts composed primarily of void space (Figs. 17I–17J), to linear structures made mostly of iron-rich mineral phases with no diagnostic original silica (Figs. 17K–17L). The cross-shaped structures are very rare in petrographic thin section (with approximately a count of one per thin section). Trilobite fragments are more common (up to eight counts per thin section, but varies); brachiopod fragments were also rare. Elongate tubes and round voids were very common, garnering a count of nearly 90 per thin section in both *Brooksella* and concretions.

**Figure 18:**
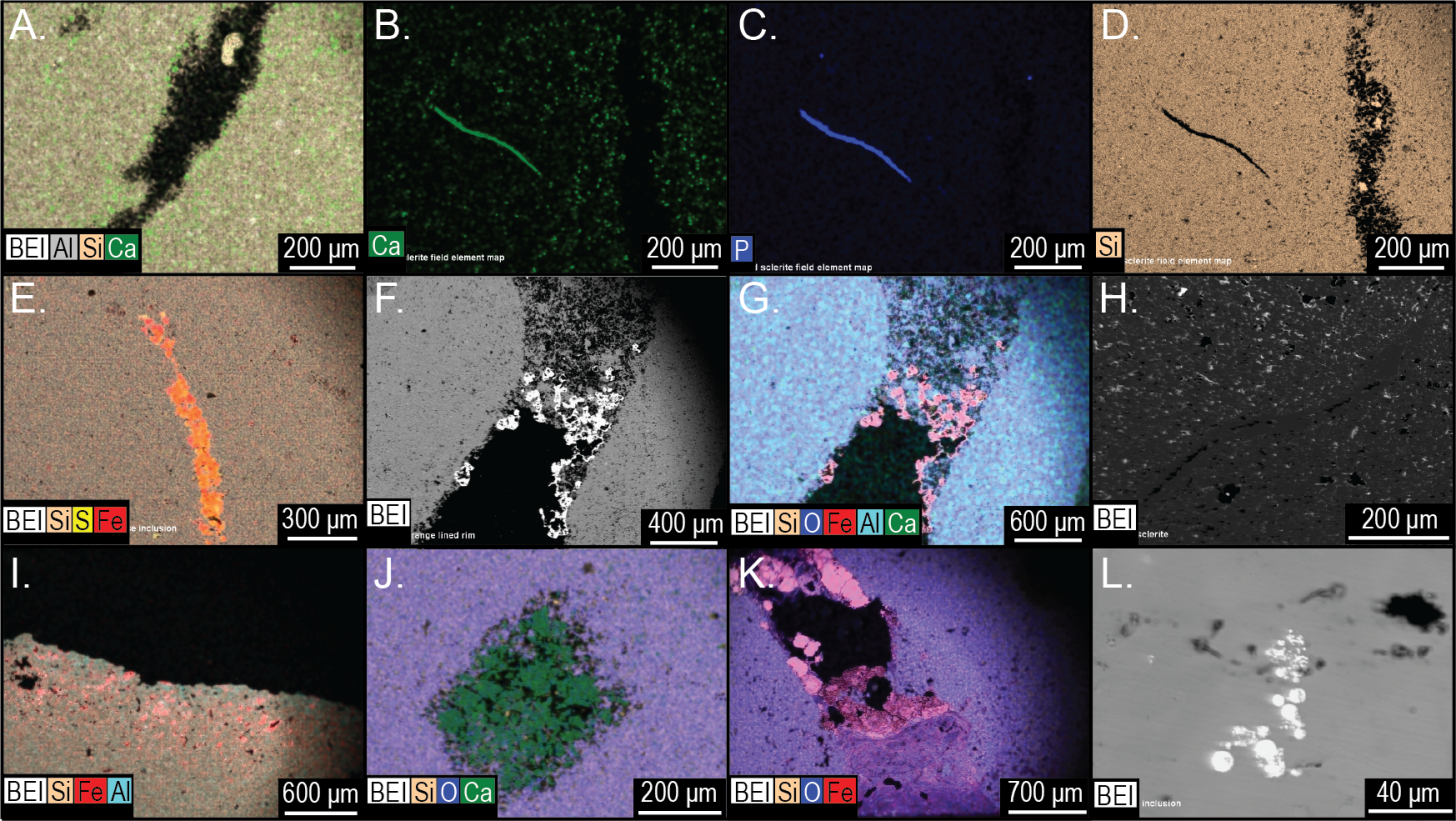
Element and backscatter electron maps of internal features of two siliceous concretions and one carbonate concretion. (A) Partial void and aluminous silica composition of the groundmass. (B–D) solid curved feature rich in phosphorous and calcium but depleted in silica. (E) inclusion containing pyrite. (F–G) surface out-letting tube partly lined with iron oxides. (H) linear feature defined by void space and silica. (I) voids along the surface weathering rind. (J) partial void filled with carbonate. (K) tube that outlets to the surface that is partly lined with pyrite and clays. (L) pyrite inclusions in a carbonate concretion UGA 157. (A–G) are from sample 27 and (I–K) are from sample 126. Energy dispersive X-ray spectra of selected features in this figure presented in Fig. S3.

Siliceous concretions had an aluminous silica composition of the groundmass like *Brooksella* (Figs. 18A, 18D–18E and 18I, Fig. S2 and Fig. S3). Trilobite fragments and linear void structures present in *Brooksella* were also found in the concretions (Figs. 18B–18E and 18H). These include Al-, Ca-, and P-rich skeletal fragments (Figs. 18B–18D), pyrite and Ba-rich inclusions (Fig. 18E), and voids defined by a lack of silica (Fig. 18H). The weathering rinds of the concretions are richer in aluminum than the interior of the specimens (Fig. 18I). Partially lined voids are also present in the siliceous concretions with iron oxide (Figs. 18F–18G), calcite (Fig. 18J), pyrite (Fig. 18K), and argillite (Figs. 18I and 18K) linings. Pyrite and titanium oxide-based inclusions are also found in the carbonate concretion (Fig. 18L).

## Discussion

### Orientation and occurrence of *Brooksella* in Conasauga shale beds

If *Brooksella* is a hexactinellid sponge, it is very rare in shale beds compared to concretions and its orientation indicates that the central depression (previously interpreted as the osculum) and lobes are mostly facing downward into the sediment, either as a once-living sponge or oriented in that position after death. Further, *in situ* concretions adjacent to *Brooksella* in the same bed are generally oriented with their more convex portion upward, similar to *Brooksella*. Both appear to deform the laminae around them. The shale beds were not overturned in this region, so their orientations represent how they were preserved or formed.

Ciampaglio et al. (2006) suggested that the convex side (bottom side of the cup-shaped *Brooksella*) is oriented downward in the sediment and that the concave side with central depression points upward suggestive of feeding mode for the sponge. They stated that their orientation was opposite of Walcott (1898), who had his medusoid *Brooksella* oriented with its lobes downward in the sediment, with the smooth top part of the bell oriented upward. While we do not agree that *Brooksella* is a medusoid, we do agree with Walcott’s interpretation of *Brooksella*’s orientation, with the lobes pointing downward in the sediment as corroborated by their field orientation in the shales.

While the entire body of a sponge can act as a filter (Kowalke, 2000), having *Brooksella*’s lobes and central depression (the osculum) facing downward into the sediment does not permit feeding nor efficient water flow through the putative oscula and radial chambers, especially in clay-dominated environments. Increased clay particles decrease filtration efficiency for hexactinellids that live oriented above the sediment-water interface (Kowalke, 2000); and all known living hexactinellid sponges live usually rooted in the sediment, and their filtering structure lies above the sediment-water interface (Hooper & Van Soest, 2002). Therefore, the orientation of *Brooksella* seemingly upside down in the sediments calls into question whether it is a sponge.

### Is *Brooksella* a hexactinellid sponge?

### External and internal sponge characteristics reexamined

Ciampaglio et al. (2006) cite that *Brooksella* is exceptionally preserved in 3-D as a cup-shaped fossil in profile. They also cite the presence of cross-shaped siliceous spicules on the outer surface which are characteristic of the hexactinellid family Protospongiidae to which they assigned *Brooksella*. They also observed the following as evidence for a sponge affinity for *Brooksella*: white spicules on the cross-sectioned polished surface; crater-like ostia on the outer surface; chamber openings on their lobe tips; internal radial canals in each lobe; and a spongocoel. However, we found that there were no stauractin siliceous spicules on the outer surface of *Brooksella* or white spicules on the cross-sectional surface. Rather, the white appearing structures are actually round voids and tubes and not sponge spicules. We did find in some of our petrographic thin sections at least one cross-shaped tube-like structure, but they cannot reliably be assigned to stauractines as they are poorly preserved (Figs. 17I–17J).

Walcott examined many thin sections of *Brooksella* and failed to find any evidence of spicules and suggested, if they were there, they were destroyed during fossilization (Walcott, 1898, p. 21). However, he did mention that casts of spicules occur on a few nodules but does not explicitly state what shape the casts are and if they were found on *Brooksella*. Importantly, both our CT and *μ*CT data indicate that *Brooksella* have a dense outer region, corresponding to an iron-oxide aluminous weathering rind. These scans do not show arranged spicules in this outer surface as would be present in protospongiids. Such a loose framework could be obscured by diagenetic processes, but there were also no spicules deeper within the specimens, where they would likely be better preserved.

We also could not find any crater-like ostia on the outer surface of *Brooksella.* Instead, we found lichen growing on the surfaces, and when the lichen were removed, they left small round nearly microscopic bioeroded pits, which possibly could be mistaken for ostia. These surface lichen pits were not connected to any internal chambers based on our thin-section, CT, and *μ*CT analyses.

The lobes of our *Brooksella* did not have terminal openings. There were also no radial canals attached to such openings that connected to a central depression and no internal lumen consistent with a spongocoel. Walcott’s images rarely depict a *Brooksella* with putative radial canals (refer to Walcott’s *Brooksella* images reprinted in Fig. 1D), and those that he thought had them at the tips of the lobes could represent taphonomic effects (Figs. 1B–1C). He noted that “not one in a hundred of the fossil specimens” had any structure within the bodies, except for some samples from one site which he doesn’t describe. However, darkened regions within *Brooksella* and concretions can occur, but not always, and these regions vary in size and shape depending on which serial cross-section is examined. None of these inner darker regions penetrated into the lobes or appeared to form a spongocoel that connected to the lobes or central depression (Fig. 14A–14B). Further, no distinct radial lobes were seen in composite 3-D reconstructions of *Brooksella* or concretions from CT and *μ*CT scans (refer to Figs. 15 and 16). That is, no internal structures appear to represent a central cavity like a spongocoel with radial canals emanating from a central region. Rather, both *Brooksella* and concretions appear to have randomly oriented internal burrow- and tube-like structures and mineralized fossil fragments. Additionally, had radial canals corresponding to lobes as described by Ciampaglio et al. (2006) been present, this would have been inconsistent with the proposed protospongiid identity, as protospongiids have thin walls and lack internal structures like radial canals or chambers (Botting & Muir, 2018).

Our *Brooksella* and silica concretions were found to commonly contain round voids and what we refer to as tubes as we do not know for certain how these structures formed (Figs. 13, 14, 15 and 16). Some larger round voids and tubes are most likely bioerosion from tree roots, and these often have an iron-oxide rind and infill (Figs. 13A and 13C), but others were much smaller (Figs. 13B and 13D). These smaller tubes can have a vertical and horizontal orientation within *Brooksella* and concretions and can vary in width and shape (Figs. 16D and 16H). Voids can be parts of tubes cut in half during thin- and *μ*CT-section analyses. We speculate that these smaller structures are likely formed by bioerosion (straight-edged tube walls) or burrows (diffuse tube walls; Figs. 16D, 16H, 16L and 16P). In Walcott (1898, p. 12), Professor Iddings examined thin sections of *Brooksella* and also noted “numerous gas pores” as part of the siliceous nodule composition, but neither Walcott nor Iddings considered those structures further. No fossil sponges, whether hexactinellid or not, are reported to have these tubes and voids.

The voids and tubes can be lined with framboidal pyrite, barite, calcium carbonate, or clay (Figs. 17C–17D, 17E–17G; Figs. 18J–18K). Framboidal pyrite is reported from algal borings in Ordovician brachiopods (Kobluk & Risk, 1977), which suggests early diagenesis just below the sediment-water interface in the bacterial sulfate reduction zone. Similarly, barite can be an early diagenetic mineral, which can form in the early stages of concretionary growth (Bojanowski et al., 2019). Early diagenesis is suggested because barite dissolves if sulfate is reduced during deep burial and if it is not protected within a microcrystalline concretion (Bojanowski et al., 2019). Calcium carbonate infilling of tubes may originate from partial dissolution of trilobite and other carbonate fossil fragments within *Brooksella* and concretions or from later diagenetic fluids.

### Size relationship between *Brooksella* and concretions

There was a significant difference in the grand geometric mean sizes among our *Brooksella* and concretions as well as Walcott’s *Brooksella*. The mean size of our concretions was slightly larger than our *Brooksella*, but both concretions and our *Brooksella* were much larger than Walcott’s *Brooksella*, suggesting that his samples were likely picked for a particular size range to be shown at natural size for comparison in his 1898 monograph. Overall, the maximum size constraints for *Brooksella*’s growth and that of concretions are different.

Nevertheless, Model II regressions indicate that size relationships in our *Brooksella* compared to concretions were not different and indicated that the maximum and minimum diameter among *Brooksella,* concretions and Walcott’s *Brooksella* were moderately to well correlated. While Walcott’s *Brooksella* were highly correlated (*r* = 0.94) and 89% of the data variation was explained by the slope, for our *Brooksella* and concretions they were only moderately correlated (*r* = 0.57 and *r* = 0.52, respectively), with only half of the data explained by the Model II regression slope. This finding indicates that not only were our *Brooksella* much more variable in diameter than those depicted in Walcott’s 1898 monograph but also that our *Brooksella* and concretions were both variable in shape and also grow similarly, although concretions can grow to a larger size.

Hexactinellid sponges exhibit age-related patterns of growth, displaying either linear growth or linear until a plateau is reached during growth (Leys & Lauzon, 1998; Botting, 2003). While growth in *Brooksella* appears somewhat linear, its growth was no different from concretionary growth, and half the data was not explained by the Model II regression slopes for both specimen types. Additionally, there was no trend or correlation for maximum lobe size to overall body size in *Brooksella,* thus lobes are not growing larger as body size increases. Further, the number of lobes did not demonstrably increase with size for *Brooksella,* given the number of lobes that *Brooksella* can have. Therefore, these results are not consistent with the general pattern of hexactinellid growth. Given the observed differences between expected sponge characteristics and the composition and microstructure of *Brooksella* that is shared with concretions, we do not accept the hexactinellid sponge identity.

### Non-sponge interpretations of *Brooksella*

### Trace fossil affinities

*Brooksella* is attributed to several different trace fossils, but usually it is thought to represent a probing-style feeding burrow. Fürsich & Kennedy (1975) postulated that *Brooksella* represented the trace fossil *Dactyloidites*, a view that was echoed by Rindsberg (2000). This identity is consistent with the general shape and orientation of many *Brooksella* samples, but *Brooksella* lacks the central tube and spreiten of *Dactyloidites.* Furthermore, radial probing actions fail to explain the tubular features observed within *Brooksella*. Similarly, *Asterosoma,* an ichnogenus of probing burrows (Seilacher, 2007), is thought to be a *Brooksella*. Certain types of *Asterosoma* display radial lobes, although these lobes are clearly distinct from *Brooksella* in their fusiform shape, often branching arrangement, and surficial cracking. The earliest *Asterosoma* are known from the Devonian, in sandstone. They have backfilled lobes, are oriented stratigraphically with the convex side of lobes upwards, and have central connecting tubes—all of which is in contrast to the shale-hosted, non-backfilled, stratigraphically downward-oriented lobes with no central connecting tubes in *Brooksella*. *Gyrophyllites*, another fodinichnia characterized by radial lobes, backfill, and a central tube is another possible identity for *Brooksella* that was suggested by Seilacher (2007). *Gyrophyllites* include both upward and downward probing, so the concave face can be oriented in either direction. These ichnofossils typically occur as impressions rather than in positive relief like *Brooksella*, which lack discernable back filling inside the lobes.

Schwimmer, Frazier & Montante (2012) suggested that *Brooksella* was a coprolite. However, the middle Cambrian age of *Brooksella* rules out production of such large feces by much larger organisms. Further, *Brooksella* specimens lack fecal pellets, and the interiors of *Brooksella* lack the directional orientation of similar materials in coprolites.

Other than superficial resemblance, *Brooksella*’s internal and external morphology do not match any previous described trace fossil.

### Pseudofossil affinities

Proposed identities for *Brooksella* have not been limited to those of biological origin. Through dewatering or other pressure imbalance processes, sand or other sediments can rise to the sediment surface, producing a “sand volcano”, which can be preserved as the pseudofossil *Astropolithon* (Seilacher, 2007). These features can take on lobate forms similar to *Brooksella* because remnant surficial biofilms could hold the erupted sands together long enough for lithification to occur. *Brooksella canyonensis* was first described as a cnidarian before being reevaluated as a pseudofossil produced in this manner, but the mechanism of fluid escape is unlikely to have produced *Brooksella alternata*. Fluid escape structures produce lobes that are oriented with convex sides stratigraphically upwards, while the lobes of *Brooksella* are mostly oriented stratigraphically downwards and lobes can occur on both sides in nearly half the specimens. Additionally, *Astrolopithon*-type structures typically occur *via* repeated eruption from the same radial cracks, producing an upward growing series of sediment layers. *Brooksella* lacks the horizontal layers that such a mechanism would produce. *Brooksella* also lacks a central vertical tubular feature and it is compositionally different from the surrounding sediments. Similarly, gas rising from dewatering sediments was cited as a possible mechanism for *Brooksella* formation. While this origin could account for the differing lithology as silica could precipitate where the gas bubbles reside and possibly explain the tubular features and voids, it does not account for the complex, lobate form of *Brooksella*.

### Concretion affinities

Both *Brooksella* and co-occurring siliceous concretions have similar shapes, remnant skeletal fossil components, weathering rinds, and internal composition; the only feature that concretions lack are lobes. In fact, *Brooksella* is recognized by the presence of at least two lobes given Walcott’s descriptions and our specimens (refer to Fig. 10). Concretions can overlap the size range of *Brooksella*, but their grand geometric mean size is significantly larger than *Brooksella* suggesting a limit to *Brooksella* size. Like Walcott (1898) observed, we also found that the composition for *Brooksella* is primarily silica with minor amounts of calcium carbonate, which is identical to the concretions. The composition of tube- and void-infilling barite and framboidal pyrite indicate the silica-rich *Brooksella* and concretions were likely formed during early diagenetic processes.

In both *Brooksella* and silica concretions, there was a lack of concentric zoning which a Professor Hayes also recognized for Walcott’s samples (Walcott, 1898, p. 12). Professor Hayes also noticed that some *Brooksella* had “parallel mica scales” which he surmised were part of the shale laminations (Walcott, 1898, p. 13), suggestive of replacive growth in carbonate concretions (after Gaines & Vorhies, 2016). However, we found no interior sedimentary layers or mica inside *Brooksella* or concretions, but shale laminations were deformed around both. Thus, we would argue that the concretions and *Brooksella* likely represent a type of displacive growth seen for carbonate nodules (Gaines & Vorhies, 2016) and represent one mode of growth (Bojanowski et al., 2019). Though some concretions and *Brooksella* had a darker region in the interior that varied in shape (refer to Figs. 13 and 14), there were no definitive concentric growth regions suggestive of concentric growth concretions (Raiswell et al., 1988; Gaines & Vorhies, 2016). However, some internal tubular structures occur within the central portion (*e.g.*, Fig. 15H) of *Brooksella*, but do not correspond to lobes, and are not arranged radially. These internal tubular structures may represent burrow traces exploring the unlithified portions around the lithified concretionary nucleus as the concretion grew over a short timescale (after Kastigar, 2016).

In summation, there is no difference between *Brooksella* and concretions except for the presence of lobes. We posit that *Brooksella* be considered an early diagenetic displacive silica concretion until more evidence can be produced that it was a biogenic structure.

### Silica sources for *Brooksella* and concretions

Cambrian seas were rich in silica and were the source for primary silica, while post-Cambrian silica cycles are dominated by biological activity (Gao et al., 2020). It is postulated that during the Ediacaran and Cambrian, silica came from a variety of sources: Silica-rich hydrothermal fluids; inorganic precipitation from seawater; authigenic clay mineral formation; cyanobacteria facilitating silica precipitation; silica adsorption on organic matter; or from silica-secreting organisms (Gao et al., 2020 and references therein; Hesse, 1989; Schieber, Krinsley & Riciputi, 2000; Vorhies & Gaines, 2009; Gaines et al., 2012).

The early Paleozoic oceans were supersaturated with respect to silica compared to undersaturated modern oceans where the silica cycle is controlled primarily by diatoms and radiolarians (Gao et al., 2020). Therefore, it is suggested that siliceous-secreting sponges and radiolarians were not a major component of the silica cycle in Ediacaran and Cambrian seas (Gao et al., 2020), though other researchers attribute a decline in oceanic dissolved Si during the Ediacaran-Cambrian transition to the onset of significant sponge biosilicification (see Chang et al., 2019). Sperling et al. (2010) suggest that an abundance of Al^3+^-rich clay minerals in the Cambrian was conducive to the preservation of siliceous spicules relative to the Ediacaran. Concretions and *Brooksella* from the Conasauga Formation have similar compositions of silica with minor amounts of clay indicative of a clastic source for the silica, but adsorption onto organic matter can also not be ruled out. It was postulated that the silica-rich *Brooksella* was derived from remobilized biogenic silica from a presumably sponge-rich time around decaying organic matter associated with microbial and/or fungal biofilms (Ciampaglio et al., 2006; Schwimmer & Montante, 2007; Kastigar, 2016). Siliceous-spicule secreting hexactinellid sponges were becoming more common in the middle Cambrian (Finks, 2003; Reid, 2009), and a combination of both inorganically precipitated silica and biogenic silica cannot be ruled out as the source of silica for the concretions, including *Brooksella*.

## Conclusions

In the century since its original description by Walcott (1896) and Walcott (1898), star-shaped siliceous nodules known as *Brooksella alternata* from the middle Cambrian Conasauga Formation, Southeastern USA, have raised numerous questions for researchers of the Cambrian. *Brooksella*’s long history of description and reevaluation from a jellyfish to a sponge or gas bubbles to trace fossils, mirrors the evolving understanding of life and environments that shaped the Cambrian seas and highlights one of the most persistent challenges in the study of early complex life—the difficulty of distinguishing life from non-life.

Although *Brooksella* and all its *Brooksella*-like forms were synonymized as *Brooksella alternata*, a hexactinellid sponge of the Protospongiidae family (Ciampaglio et al., 2006), we found no sponge-like diagnostic characteristics on either the external surface or internal regions of *Brooksella*. “Ostia” were likely lichen-etched pits on the surface of *Brooksella*, as modern lichen was common on *Brooksella* and concretions. Spicules were not present on either *Brooksella* surfaces or their interiors, although very rare, roughly cross-shaped ghosts in both concretions and *Brooksella* may have represented a stauractine at one time, but there was no definitive elemental analysis that supports these ghosts as being siliceous spicules. “White spicules” observed by Ciampaglio et al. (2006) on polished cross-sections were abundant, round voids and tubes that appeared light colored but were not siliceous spicules. Walcott (1898) also did not find spicules after examining hundreds of *Brooksella,* but observed some on the external surfaces of some concretions. A central depression (“osculum”) was not common on *Brooksella*, and an internal spongocoel did not occur. Some concretions, and rarely *Brooksella,* had a diagenetic somewhat central region that could be conflated as a spongocoel, but this structure varied in shape depending on how it was cut and was not connected to any radial canals or chambers. *Brooksella’*s external lobes had no radial canals in the interior nor were radial canals visible in CT scans or thin sections.

Importantly, thin sections, CT, and µCT scans of *Brooksella* and concretions reveal tubes and voids of variable size, shape, and orientation that can pass through the entire *Brooksella* or concretion and also occur in the weathered outer rind. These tubes are not consistent with radial canals proposed for the hexactinellid affinity of *Brooksella*, or with other biological affinities. Elemental analysis indicates that these tubes and voids can be lined or filled with barite, iron oxides, framboidal pyrite and occasional clays or carbonates. The framboidal pyrite and barite suggest formation in early diagenetic marine conditions during burial in the sulfate-reducing zone, although some with iron-oxide-clay infilling represent post-depositional roots or rootlets that penetrated the *Brooksella* and concretions. Other tubes/voids could be burrowing organisms from the middle Cambrian, like Walcott (1898) observed on *Laotira* specimens (refer to Figs. 1I–1J). These structures indicate that the organic accumulations that gave rise to *Brooksella* and associated concretions were likely mined for organic matter before or during the formation of these nodules, or that the growth of these nodules preserved burrows within them but the burrows did not contribute to forming lobes in *Brooksella.* These burrows were rapidly mineralized in early diagenesis and have no relation to any previous trace fossil affinities assigned to *Brooksella* like that of *Dactyloidites*.

In summary, *Brooksella* and concretions share external weathering rinds, mineralogical composition, and internal structures; only *Brooksella* possesses external lobes and sometimes, a central depression (or protuberance). *Brooksella* lacks hexactinellid sponge-defining characteristics and shares more similarities with concretions from the Conasauga Formation. Although *Brooksella* has numerous proposed identities (Fig. S1), the bulk of its characteristics are consistent with concretions. Therefore, from the sum of its parts, we suggest that *Brooksella* be considered a pseudofossil until proven otherwise, and the hypothesis that these sponges contributed biogenic silica to the exceptional preservation of the middle Cambrian Conasauga Lagerstätte needs to be reevaluated in light of the supersaturated silica-rich seas from this time period, which could have abiogenic or microbial sources. Future work on sponge biomarkers and silica stable isotopes (*δ*^30^Si) on well-preserved specimens will hopefully settle the origin of this silica and the biogenicity of *Brooksella*.

##  Supplemental Information

10.7717/peerj.14796/supp-1 Table S1 Major characteristics of *Brooksella* and *Brooksella*-like fossils as described by Walcott (1995; 1896) and (Ciampaglio et al., 2006), for the Conasauga Formation “–” indicates information not available.

10.7717/peerj.14796/supp-2 Table S2 *Brooksella* measurements If cells are empty, no measurements were taken or could be taken. No. = abbreviation for number (as in counts).

10.7717/peerj.14796/supp-3 Table S3 Concretion measurements

10.7717/peerj.14796/supp-4 Figure S1 Comparison of purported identities for *Brooksella alternata* Resser CE. 1938. Cambrian system (restricted) of the southern Appalachians. New York: The Geological Society of America.

10.7717/peerj.14796/supp-5 Figure S2 X-Ray diffractograms of two powdered *Brooksella* and two silica concretions These specimens have nearly identical mineral composition, which is predominantly silica, with some calcite.

10.7717/peerj.14796/supp-6 Figure S3 Energy dispersive X-ray spectra of features in Figure 18. (A) Electron beam scatter spectra of the dense feature shown in Fig 18E siliceous concretion sample 27. Note the presence of Fe, Ba, and S. (B) EBS spectra of a dense inclusion from siliceous concretion sample 126. Note the Ti peak. (C) EBS spectra of pyrite inclusions in a Conasauga carbonate concretion sample 157 shown in Fig 8L. Note the Fe and S peaks. (D) EBS spectra of a dense inclusion from the same carbonate concretion. Note the Ti peaks.

## Additional Information and Declarations

### Field Study Permissions

The following information was supplied relating to field study approvals (*i.e.*, approving body and any reference numbers): Our field collection complied with Alabama’s laws regarding fossil collection, which do not require permitting for this type of collection. Alabama’s laws restricting fossil collection apply to certain kinds of fossils (*Basilosaurus*), fossils collected from specific state-protected sites (*e.g.*, the Stephen C. Minkin Paleozoic Footprint Site), and from public sites where the state limits access to the lands (*e.g.*, Cedar Lake Reservoir). The collection of *Brooksella* material from the publicly accessed and unimproved shores of low-level Weiss Lake does not meet any of these restriction criteria.

### Data Availability

The following information was supplied regarding data availability: The raw data used in the study are available in Table 1 (describing field occurrence of samples), and Tables S2 and S3 (which contain the morphological measurements of the Brooksella and silica concretions respectively). The CT and uCT image stacks used in 3D reconstruction in this article are available at Morphosource (Project ID: 000436718, Brooksella and silica concretions):
https://doi.org/10.17602/M2/M491416;
https://doi.org/10.17602/M2/M491376;
https://doi.org/10.17602/M2/M491552;
https://doi.org/10.17602/M2/M491367;
https://doi.org/10.17602/M2/M491674;
https://doi.org/10.17602/M2/M491687;
https://doi.org/10.17602/M2/M491682;
https://doi.org/10.17602/M2/M491670;
https://doi.org/10.17602/M2/M491666;
https://doi.org/10.17602/M2/M491662;
https://doi.org/10.17602/M2/M491658;
https://doi.org/10.17602/M2/M491653;
https://doi.org/10.17602/M2/M491648;
https://doi.org/10.17602/M2/M491643;
https://doi.org/10.17602/M2/M491548;
https://doi.org/10.17602/M2/M491542;
https://doi.org/10.17602/M2/M491532;
https://doi.org/10.17602/M2/M491528;
https://doi.org/10.17602/M2/M491420;
https://doi.org/10.17602/M2/M491411;
https://doi.org/10.17602/M2/M491407;
https://doi.org/10.17602/M2/M491400;
https://doi.org/10.17602/M2/M491396;
https://doi.org/10.17602/M2/M491388;
https://doi.org/10.17602/M2/M491384;
https://doi.org/10.17602/M2/M491380 and
https://www.morphosource.org/projects/000436718?locale=en.
