# Supplementary material for: Is the middle Cambrian Brooksella a hexactinellid sponge, trace fossil or pseudofossil?
Source: PeerJ. 2023 Feb 24;11:e14796. doi: 10.7717/peerj.14796 (PMC9969855; doi:10.7717/peerj.14796)
Supplement: Table S1 — “–” indicates information not available. [file peerj-11-14796-s001.docx]

Table S1. Major characteristics of *Brooksella* and *Brooksella*-like fossils as described by Walcott (1995; 1896) and Ciampaglio et al., 2006, for the Conasauga Formation. “--” indicates information not available.

| Genus and Species Level | Taxonomic Designation | Size range (cm)/Average size (cm) | Top surface (exumbrella) | Bottom surface (oral side; subumbrella) | Number of lobes | Lobe characteristics | Other features/comments | Reference |
| --- | --- | --- | --- | --- | --- | --- | --- | --- |
| *Brooksella*  Genus | Body fossil--Medusa  (Order Scyphomedusae; Family Brooksellidae) | -- | Lobate umbrella which is dorsally convex | -- | 6, 7, 12 | Radial canal in each lobe, as well as in interradial lobes if present | No tentacles; no central oral opening but oral plate is quadripartite with four oral arms | Walcott 1895; 1896 |
| *Brooksella alternata* (Type species) | Body fossil--Medusa  (Order Scyphomedusae; Family Brooksellidae) | 1–5/4 | Contains lobes, dominant character in all specimens | Oral disk with ring about the central disk; strong ribs radiate from the center to each principle lobe of top surface; but Walcott later states that no trace of oral opening had been seen | 6–20 or more; most have 5 and 7 lobes or 6 and 8 lobes | Can have extra lobes in between the main lobes; radial canals extend into each lobe and are large compared to lobe size; lobes unite in center giving a true radiate appearance | “Variation too great that a brief specific diagnosis is of little valiue”; regular to irregular-lobed forms. No regular form, most irregular.  No tentacles observed; gastrovascular cavity (based on actual and theoretical information), with radial canals; oral arms; buccal stomach inferred from collapse of central disk | Walcott 1895; 1896 |
| *Brooksella alternata* | Body fossil--  Porifera  (Class Hexactinellida; Order Reticulosa; Family Protospongiidae) | 1–10/not reported | Concave side with central depression pointing upward | Convex side with central protrusion pointing downward | Commonly 4–7 but could have more | Radial lobes separated by radial grooves;  lobes often terminate in a small opening; radial internal cavities occupy lobes | Often a central opening inferred to be an osculum on one side; crater-like structures on external surface inferred to be ostia; have siliceous 4-rayed spicules on the external surface and surface of cross sections as white-colored spicules; “regular radial form, surface and internal features consistent with organic origin;” no backfill structures were found indicative of burrowing organism like teichichnoid | Ciampaglio et al., 2006 |
| *Brooksella confusa* | Body fossil--Medusa  (Order Scyphomedusae; Family Brooksellidae) | -- | -- | No visible central opening | Differs from *B. alternata* by the number of lobes of subumbrella;  a confused pile of interlobate lobes on ventral surface | Lobes do not join at center; have tendency to join near that center or a double center | Assumed lobes (“arms”) brought food right to stomach in central axis area | Walcott 1895; 1896 |
| *Laotira cambria* | Body fossil--Medusa  (Order Scyphomedusae; Family Brooksellidae) | 1.5 –8/5 | Outer surface exactly like *Brooksella*; various shapes from round to flat | No central opening; in some, similar to *B. alternata;* most subumbrella lobes do not meet at center; many samples show no regularity | 4–7 to 12 or more lobes on outer surface; some have 20 lobes; large number of compound lobes | Simple radial canal in each lobe and in compound (interradial) lobes attached to central axis | No tentacles; intermediate between *B. alternata* and *B. confusa;* has radial canals in simple forms, but others irregular canals, often not well preserved; 30–34 radial canals terminate at central area, likely a central stomach | Walcott 1895; 1896 |
