# Supplementary material for: Is the middle Cambrian Brooksella a hexactinellid sponge, trace fossil or pseudofossil?
Source: PeerJ. 2023 Feb 24;11:e14796. doi: 10.7717/peerj.14796 (PMC9969855; doi:10.7717/peerj.14796)
Supplement: Table S2 — If cells are empty, no measurements were taken or could be taken. No. = abbreviation for number (as in counts). [file peerj-11-14796-s002.docx]

Table S2. *Brooksella* measurements. If cells are empty, no measurements were taken or could be taken. No. = abbreviation for number (as in counts).

| Specimen ID | Height (mm) | Length (mm) | Width (mm) | No. of top lobes | No. of bottom lobes | Lobe Length (mm) | Lobe Width (mm) | Surface pit Diameter (mm) | Central depression Width (mm) |
| --- | --- | --- | --- | --- | --- | --- | --- | --- | --- |
| 59 | 15.03 | 26.82 | 27.73 | 0 | 0 |  |  |  |  |
| 1.1 | 18.22 | 36.48 | 67.6 | 0 | 5 | 12.94 | 14.58 |  |  |
| F2.2 | 14.4 | 36.72 | 48.76 | 0 | 6 |  |  |  |  |
| S6.4 | 13.94 | 34.6 | 39.84 | 0 | 9 | 13.18 | 7.04 |  |  |
| M6.4 | 12.88 | 30.99 | 61.34 | 0 | 5 | 11.97 | 7.61 |  | 5.52 |
| F6.3 | 15.66 | 38.77 | 42.86 | 2 | 8 | 17.64 | 10.38 |  |  |
| 4 | 16.29 |  | 43.07 | 3 | 1 | 18.16 | 15.03 |  |  |
| WSL2.AL17b | 15.28 |  | 24.40 | 3 | 0 | 11.69 | 7.63 |  |  |
| DCC1 | 14.38 |  | 54.37 | 4 | 0 | 17.45 | 11.52 |  |  |
| F6.4 | 23.7 | 41.72 | 63.37 | 4 | 0 | 15.3 | 8.79 |  |  |
| F6 | 14.83 | 6.85 | 34.07 | 4 | 0 | 22.15 | 5 |  | 2.26 |
| F6.B | 14.34 | 5.23 | 38.68 | 4 | 0 | 18.01 | 6.14 |  | 2.46 |
| 53 | 17.62 |  | 32.97 | 5 | 0 | 13.80 | 12.52 |  | 6.41 |
| 55 | 23.37 |  |  | 5 | 0 | 25.40 | 12.94 |  |  |
| 95 | 28.48 | 45.94 | 59.01 | 5 | 2 | 25.57 | 25.79 |  | 8.56 |
| 124 | 17.78 | 39.67 | 50.69 | 5 | 0 | 51.01 | 15.02 |  |  |
| 152 | 15.72 | 30.80 | 51.02 | 5 | 0 | 16.60 | 8.34 |  |  |
| LSV1.AL2 | 21.35 | 42.02 | 48.87 | 5 | 0 | 18.53 | 11.34 | 1.77 | 4.94 |
| WSL2.AL7 | 15.19 | 28.28 | 45.09 | 5 | 5 | 27.30 | 12.94 | 2.67 | 3.96 |
| WSL2.AL16 | 20.72 | 37.21 | 46.56 | 5 | 0 | 19.19 | 10.86 |  | 5.44 |
| F5.5 | 18.3 | 38.56 | 46.94 | 5 | 5 | 18.18 | 9.68 |  |  |
| F6.11 | 23.82 | 45.08 | 64.09 | 5 | 5 | 23.92 | 16.73 |  |  |
| F2 | 24.47 | 43.12 | 59 | 5 | 5 | 24.55 | 20.87 | 1.53 |  |
| 5 | 14.49 | 34.45 | 45.24 | 6 | 5 | 18.80 | 18.63 |  | 7.67 |
| 1 | 18.56 | 33.61 | 46.57 | 6 | 8 | 19.63 | 16.14 |  | 6.89 |
| 2 | 17.80 | 32.88 | 46.18 | 6 | 5 | 19.90 | 20.28 |  |  |
| 102 | 28.47 | 43.23 | 58.68 | 6 | 0 | 26.76 | 8.83 |  | 2.97 |
| 98 | 32.54 | 20.90 | 38.16 | 6 | 2 | 14.92 | 11.98 | 1.24 |  |
| WSL2.AL5 | 17.71 | 23.15 | 32.47 | 6 | 5 | 13.51 | 10.82 |  | 5.59 |
| WSL2.AL21 | 16.10 | 39.69 | 47.79 | 6 | 0 | 18.50 | 19.87 | 1.41 | 7.25 |
| F1.1 | 19.69 |  | 53.22 | 6 | 5 | 12.87 | 9.74 | 0.94 |  |
| F1.3 | 19.07 | 37.79 | 49.22 | 6 | 7 | 27.27 | 16.67 |  | 6.65 |
| F6.9 | 15.88 | 22.18 | 29.39 | 6 | 6 | 11.62 | 6.42 |  |  |
| F6.C | 14.54 | 6.92 | 27.67 | 6 | 0 | 14.28 | 4.54 |  |  |
| 118b | 11.81 | 28.39 | 31.87 | 7 | 4 | 13.42 | 8.33 |  |  |
| 121 | 14.78 | 28.69 | 48.52 | 7 | 3 | 12.56 | 21.35 |  |  |
| 155 | 12.75 | 23.43 | 38.74 | 7 | 2 | 15.98 | 18.09 |  | 10.33 |
| LSV1.AL1 | 23.40 | 43.38 | 56.14 | 7 | 0 | 20.72 | 16.11 |  | 8.56 |
| WSL2.AL1 | 18.11 | 39.83 | 42.30 | 7 | 4 | 15.11 | 15.73 |  |  |
| WSL2.AL10 | 18.41 | 39.39 | 62.25 | 7 | 0 | 19.52 | 8.63 | 1.42 |  |
| F6.1 | 9.07 | 25.59 | 40.4 | 7 | 0 | 22.95 | 9.54 |  | 7.04 |
| F6.2.B | 14.39 | 49.03 | 59.27 | 7 | 7 | 26.54 | 3.08 | 0.39 |  |
| F3 | 18.77 | 51.55 | 65.48 | 7 | 7 | 33.27 | 15.74 | 1.64 |  |
| 120 | 14.78 | 34.93 | 53.20 | 8 | 0 | 22.15 | 6.72 |  |  |
| 122 | 19.16 | 31.40 | 50.58 | 8 | 0 | 18.97 | 15.67 |  | 8.43 |
| 20 | 14.48 | 29.34 | 44.67 | 8 | 0 | 19.39 | 16.57 |  |  |
| WSL2.AL9 | 15.94 | 29.92 | 34.61 | 8 | 8 | 14.29 | 11.35 |  | 2.84 |
| WSL2.AL14 | 18.66 | 48.43 | 60.05 | 8 | 10 | 22.75 | 20.49 |  |  |
| WSL2.AL15 | 17.80 | 32.80 | 47.78 | 8 | 5 | 14.59 | 8.06 | 1.21 |  |
| WSL2.AL16b | 19.22 | 40.22 | 48.40 | 8 | 0 | 16.17 | 11.50 | 1.78 |  |
| WSL2.AL17 | 23.49 | 41.23 | 50.59 | 8 | 0 | 17.50 | 11.36 |  | 2.07 |
| WSL2.AL19 | 15.15 | 37.35 | 44.27 | 8 | 7 | 19.87 | 16.33 |  | 3.63 |
| WSL2.AL20 | 15.90 | 38.53 | 52.62 | 8 | 0 | 13.50 | 5.03 |  | 5.37 |
| 3 | 16.57 | 33.36 | 48.48 | 9 | 6 | 19.57 | 19.06 |  |  |
| 7 | 14.92 | 34.83 | 43.80 | 9 | 0 | 18.14 | 15.37 |  |  |
| WSL2.AL2 | 23.23 | 39.73 | 56.11 | 9 | 6 | 21.56 | 12.38 |  |  |
| WSL2.AL3 | 18.39 | 41.33 | 50.17 | 9 | 0 | 15.38 | 8.95 |  | 4.4 |
| WSL2.AL6 | 14.36 | 29.03 | 34.14 | 9 | 0 | 12.45 | 10.39 | 2.07 | 3.62 |
| WSL2.AL8 | 15.16 | 37.79 | 53.87 | 9 | 6 | 19.84 | 12.90 |  | 3.23 |
| WSL2.AL22 | 15.32 | 51.86 | 67.19 | 9 | 0 | 20.88 | 9.27 |  |  |
| F6.2 | 14.1 | 42.84 | 57.38 | 9 | 12 | 19.25 | 16.5 |  |  |
| 6 | 18.41 | 39.17 | 44.26 | 10 | 0 | 18.61 | 18.64 |  |  |
| 54 | 16.61 | 39.16 | 59.49 | 10 | 7 | 22.65 | 13.72 | 2.02 | 3.23 |
| 56 | 15.15 | 38.18 | 45.86 | 10 | 0 | 20.66 | 14.28 | 1.19 |  |
| F1.2 | 20.66 | 46.72 | 58.29 | 10 | 9 | 22.41 | 11.66 |  |  |
| 8 | 19.86 | 42.00 | 47.70 | 11 | 0 | 14.69 | 9.06 | 1.16 |  |
| 17 | 18.05 | 39.50 | 69.10 | 11 | 9 | 31.71 | 17.84 |  | 7.81 |
| WSL2.AL12 | 21.03 | 39.50 | 44.71 | 11 | 0 | 14.39 | 7.65 |  |  |
| WSL2.AL13 | 10.43 | 33.58 | 42.72 | 11 | 6 | 14.35 | 8.84 | 1.24 |  |
| WSL2.AL18 | 19.20 | 60.20 | 73.59 | 11 | 0 | 18.91 | 9.10 |  |  |
| 57 | 15.81 | 35.93 | 46.51 | 12 | 6 | 19.13 | 13.24 |  |  |
| 118 | 14.56 | 40.46 | 50.48 | 12 | 4 | 17.68 | 6.43 |  | 13.3 |
| WSL2.AL11 | 16.17 | 45.25 | 58.55 | 12 | 7 | 18.74 | 8.11 |  | 5.56 |
| 153 | 23.82 | 47.31 | 78.00 | 13 | 0 | 19.56 | 12.12 |  |  |
| WSL2.AL4 | 15.89 | 40.90 | 56.46 | 13 | 0 | 12.27 | 7.13 |  |  |
| 119 | 17.50 | 37.34 | 43.74 | 14 | 0 | 18.68 | 8.15 | 2.07 |  |
| F1.4 | 19.32 | 40.46 | 53.96 | 15 | 9 | 27.53 | 7.55 |  |  |
