# Supplementary material for: Is the middle Cambrian Brooksella a hexactinellid sponge, trace fossil or pseudofossil?
Source: PeerJ. 2023 Feb 24;11:e14796. doi: 10.7717/peerj.14796 (PMC9969855; doi:10.7717/peerj.14796)
Supplement: Table S3 [file peerj-11-14796-s003.docx]

Table S3. Concretion measurements.

| Specimen ID | Minimum Diameter of whole specimen (mm) | Maximum Diameter of whole specimen (mm) | Average (Central Tendency) Diameter | Ratio of Length to Width |
| --- | --- | --- | --- | --- |
| 22 | 36.51 | 52.01 | 43.58 | 0.702 |
| 41 | 43.87 | 73.14 | 56.64 | 0.5998 |
| 69 | 58.04 | 69.11 | 63.33 | 0.8398 |
| 77 | 36.37 | 45.65 | 40.75 | 0.7967 |
| 74 | 44.84 | 52.27 | 48.41 | 0.8579 |
| 71 | 51.94 | 78.26 | 63.76 | 0.6637 |
| 72 | 43.79 | 65.07 | 53.38 | 0.673 |
| 78 | 48.72 | 60.28 | 54.19 | 0.8082 |
| 65 | 50.49 | 67.45 | 58.36 | 0.7486 |
| 73 | 49.51 | 67.09 | 57.63 | 0.738 |
| 46 | 31.61 | 45.74 | 38.02 | 0.6911 |
| 42 | 25.03 | 51.31 | 35.84 | 0.4878 |
| 65.1 | 44.14 | 64.63 | 53.41 | 0.683 |
| 136 | 45.74 | 72.49 | 57.58 | 0.631 |
| 70 | 43.57 | 60 | 51.13 | 0.7262 |
| 66 | 38.55 | 64.22 | 49.76 | 0.6003 |
| 68 | 28.11 | 62.94 | 42.06 | 0.4466 |
| 25 | 31.21 | 62.55 | 44.18 | 0.499 |
| 76 | 32.04 | 45.56 | 38.21 | 0.7032 |
| 82 | 27.78 | 54.42 | 38.88 | 0.5105 |
| 75 | 40.19 | 49.11 | 44.43 | 0.8184 |
| 67 | 24.53 | 59.18 | 38.1 | 0.4145 |
| 80 | 41.37 | 44.53 | 42.92 | 0.929 |
| 83 | 35.17 | 44.4 | 39.52 | 0.7921 |
| 79 | 38.68 | 45.42 | 41.91 | 0.8516 |
| 81 | 46.2 | 67.24 | 55.74 | 0.6871 |
| F5.4 | 41.43 | 51.84 | 46.34 | 0.7992 |
| F6.5 | 33.68 | 42.62 | 37.89 | 0.7902 |
| F6.7 | 30.56 | 34.14 | 32.3 | 0.8951 |
| F6.6 | 28.52 | 29.04 | 28.78 | 0.9821 |
| F5.10 | 22.9 | 61.83 | 37.63 | 0.3704 |
| F5.9 | 49.11 | 62.02 | 55.19 | 0.7918 |
| F6.3 | 40.8 | 42.73 | 41.75 | 0.9548 |
| F5.11 | 51.41 | 69.25 | 59.67 | 0.7424 |
| F6.4 | 43.37 | 63.91 | 52.65 | 0.6786 |
| F5.7 | 23.27 | 29.18 | 26.06 | 0.7975 |
| F5.3 | 52.77 | 67.44 | 59.66 | 0.7825 |
| F5.6 | 20.85 | 33.94 | 26.6 | 0.6143 |
| F2.3 | 27.77 | 56.85 | 39.73 | 0.4885 |
| F2.1 | 21.16 | 40.97 | 29.44 | 0.5165 |
| 148 | 42.55 | 64.12 | 52.23 | 0.6636 |
| 39 | 47.46 | 72.47 | 58.65 | 0.6549 |
| 45 | 45.4 | 61.94 | 53.03 | 0.733 |
| 33 | 34.49 | 44.25 | 39.07 | 0.7794 |
| 145 | 45.01 | 76.38 | 58.63 | 0.5893 |
| 150 | 55.14 | 74.77 | 64.21 | 0.7375 |
| 149 | 35.53 | 69.85 | 49.82 | 0.5087 |
| 47 | 51.83 | 87.66 | 67.4 | 0.5913 |
| 36 | 21.36 | 25.49 | 23.33 | 0.838 |
| 137 | 41.34 | 53.87 | 47.19 | 0.7674 |
| 140 | 30.41 | 39.64 | 34.72 | 0.7672 |
| 43 | 46.7 | 75.89 | 59.53 | 0.6154 |
| 37 | 33.62 | 48.5 | 40.38 | 0.6932 |
| 34 | 31.4 | 71.06 | 47.24 | 0.4419 |
| 135 | 23.75 | 32.78 | 27.9 | 0.7245 |
| 14 | 37.98 | 73.48 | 52.83 | 0.5169 |
| 13 | 38.26 | 81.53 | 55.85 | 0.4693 |
| 15 | 53.76 | 68.81 | 60.82 | 0.7813 |
| 48 | 30.68 | 47.69 | 38.25 | 0.6433 |
| 30 | 14.99 | 19.48 | 17.09 | 0.7695 |
| 10 | 37.14 | 75.62 | 53 | 0.4911 |
| 16 | 47.74 | 58.28 | 52.75 | 0.8191 |
| 11 | 34.92 | 58.57 | 45.22 | 0.5962 |
| 23 | 39.93 | 82 | 57.22 | 0.487 |
| 12 | 29.67 | 53.82 | 39.96 | 0.5513 |
| 24 | 55.3 | 81.43 | 67.1 | 0.6791 |
| 28 | 83.18 | 102.47 | 92.32 | 0.8117 |
| 5.3 | 43.05 | 68.14 | 54.16 | 0.6318 |
| 5.4 | 39.67 | 59.19 | 48.46 | 0.6702 |
| 5.8 | 47.15 | 68.89 | 56.99 | 0.6844 |
| 1.3 | 36.7 | 66.92 | 49.56 | 0.5484 |
| 5.9 | 74.02 | 85.15 | 79.39 | 0.8693 |
| 5.16 | 35.41 | 55.69 | 44.41 | 0.6358 |
| 1.2 | 38.85 | 54.73 | 46.11 | 0.7098 |
| 5.2 | 49.81 | 61.37 | 55.29 | 0.8116 |
| 5.15 | 36.41 | 62.79 | 47.81 | 0.5799 |
| 5.14 | 63.84 | 77.57 | 70.37 | 0.823 |
| 5.7 | 44.34 | 69.05 | 55.33 | 0.6421 |
| 5.11 | 30.9 | 60.06 | 43.08 | 0.5145 |
| 5.13 | 45.52 | 61.44 | 52.88 | 0.7409 |
| 5.6 | 55.9 | 72.84 | 63.81 | 0.7674 |
| 5.10. | 38.21 | 70.49 | 51.9 | 0.5421 |
| 5.1 | 44.97 | 56.5 | 50.41 | 0.7959 |
| 1.1 | 39.21 | 67.66 | 51.51 | 0.5795 |
| 5.12 | 41.05 | 59.97 | 49.62 | 0.6845 |
| F6.8 | 21.85 | 59.85 | 36.16 | 0.3651 |
| F6.9 | 23.01 | 29.26 | 25.95 | 0.7864 |
| F6.10 | 43.82 | 82.78 | 60.23 | 0.5294 |
| M6.6 | 29.6 | 74.65 | 47.01 | 0.3965 |
| S6.5 | 33.12 | 71.2 | 48.56 | 0.4652 |
| M6.5 | 25.23 | 44.4 | 33.47 | 0.5682 |
| S6.1 | 58.97 | 94.9 | 74.81 | 0.6214 |
| M6.1 | 50.92 | 55.79 | 53.3 | 0.9127 |
| M6.2 | 45.83 | 73.3 | 57.96 | 0.6252 |
| 133 | 30.36 | 37.16 | 33.59 | 0.817 |
| 129 | 65.92 | 84.69 | 74.72 | 0.7784 |
| 32 | 35.49 | 46.36 | 40.56 | 0.7655 |
| 147 | 41.46 | 49.92 | 45.49 | 0.8305 |
| 128 | 46.2 | 74.21 | 58.55 | 0.6226 |
