# Supplementary figures and images for: Is the middle Cambrian Brooksella a hexactinellid sponge, trace fossil or pseudofossil?
Source: PeerJ. 2023 Feb 24;11:e14796. doi: 10.7717/peerj.14796 (PMC9969855; doi:10.7717/peerj.14796)

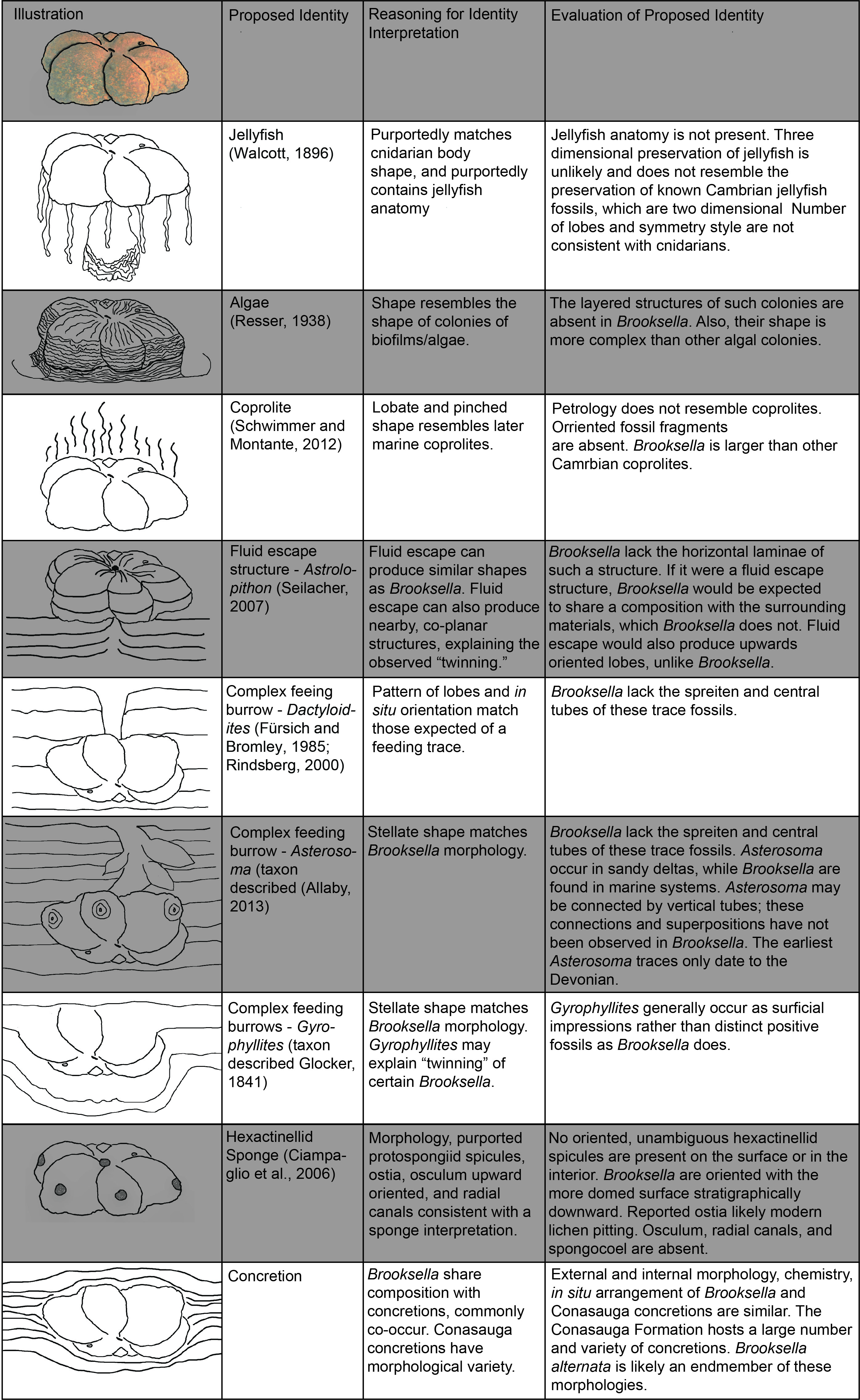

Supplement: Figure S1 — Resser CE. 1938. Cambrian system (restricted) of the southern Appalachians. New York: The Geological Society of America. [file peerj-11-14796-s004.jpg]

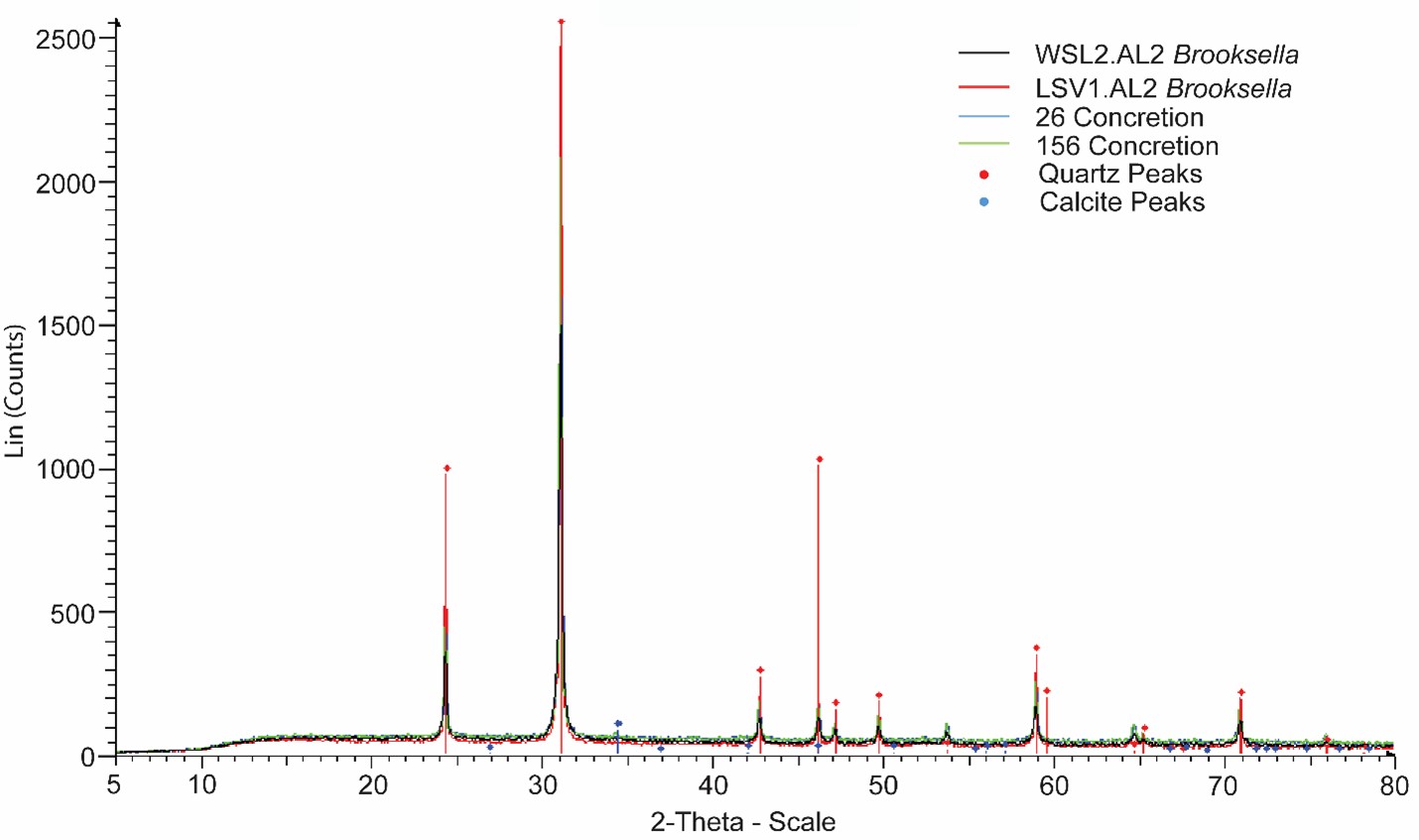

Supplement: Figure S2 — These specimens have nearly identical mineral composition, which is predominantly silica, with some calcite. [file peerj-11-14796-s005.jpg]

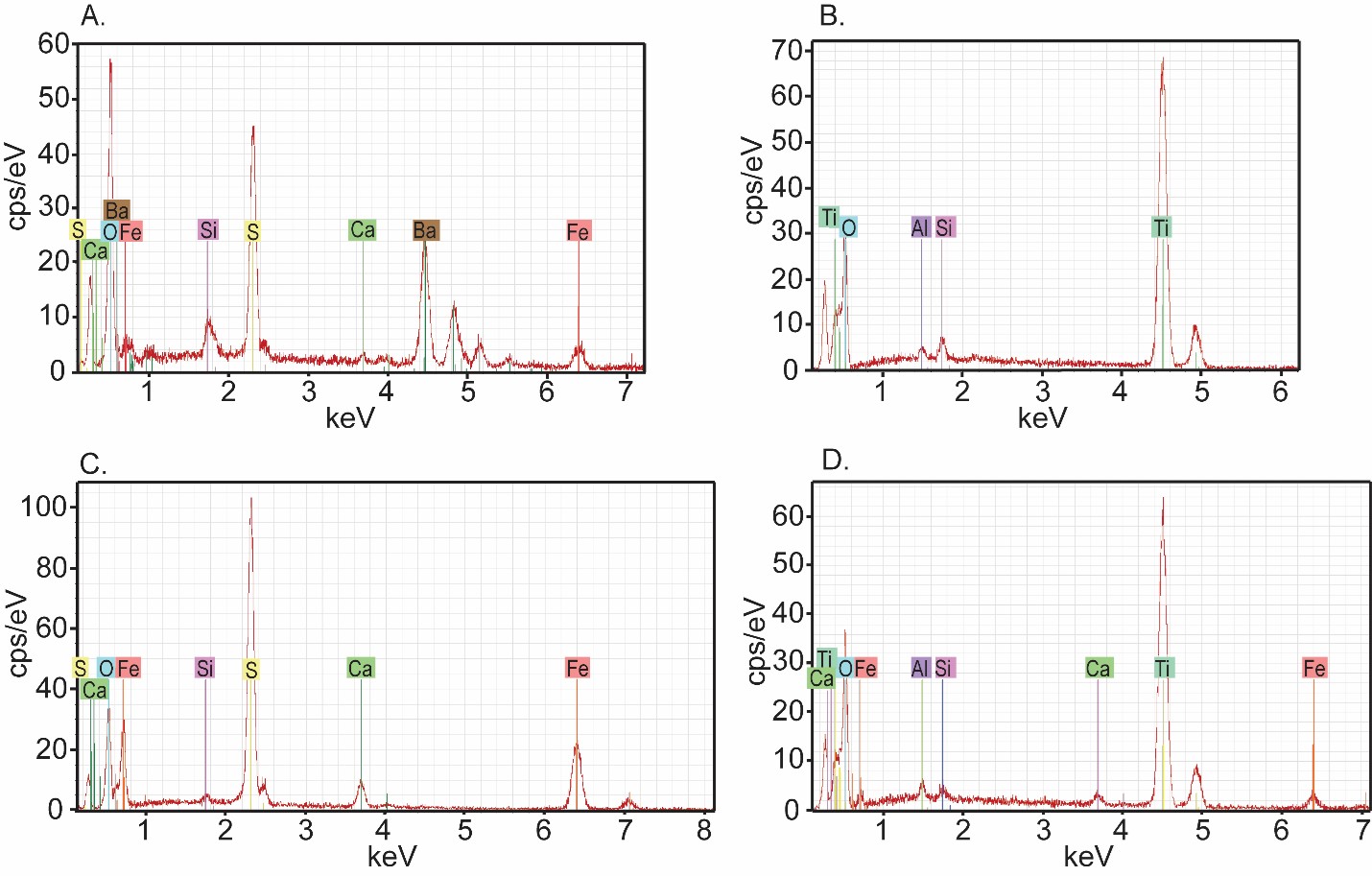

Supplement: Figure S3 — (A) Electron beam scatter spectra of the dense feature shown in Fig 18E siliceous concretion sample 27. Note the presence of Fe, Ba, and S. (B) EBS spectra of a dense inclusion from siliceous concretion sample 126. Note the Ti peak. (C) EBS spectra of pyrite inclusions in a Conasauga carbonate concretion sample 157 shown in Fig 8L. Note the Fe and S peaks. (D) EBS spectra of a dense inclusion from the same carbonate concretion. Note the Ti peaks. [file peerj-11-14796-s006.jpg]
